# Eco-Friendly Synthesis, Physicochemical Characterization, and In Vitro Biological Evaluation of Plant-Derived Bioactive-Loaded Chitosan Nanoparticles Supported by Molecular Modeling Studies

**DOI:** 10.3390/molecules31152677

**Published:** 2026-07-31

**Authors:** Ajwa, Fatma Hussain, Amer Jamil, Bilal Aslam, Piotr Weber, Jacek Nowaczyk, Alicja Nowaczyk

**Affiliations:** 1 Department of Biochemistry, University of Agriculture, Faisalabad 38040, Pakistan; ajwarana03@gmail.com (A.); fatmah@uaf.edu.pk (F.H.); amerjamil@uaf.edu.pk (A.J.); 2 Institute of Physiology and Pharmacology, University of Agriculture Faisalabad, Faisalabad 38040, Pakistan; bilal.aslam@uaf.edu.pk; 3 Institute of Physics and Applied Computer Science, Faculty of Applied Physics and Mathematics, Gdańsk University of Technology, 11/12G. Narutowicza St., 80-233 Gdansk, Poland; piotr.weber@pg.edu.pl; 4 Faculty of Chemistry, Nicolaus Copernicus University, 7 Gagarina St., 87-100 Torun, Poland; jacek.nowaczyk@umk.pl; 5 Department of Organic Chemistry, Faculty of Pharmacy, Collegium Medicum in Bydgoszcz, Nicolaus Copernicus University, 2 Jurasza St., 85-089 Bydgoszcz, Poland

**Keywords:** *Silybum marianum*, flavonolignans, phenolic compounds, chitosan nanoparticles, nanocarrier drug delivery, antioxidant activity, antidiabetic potential

## Abstract

*Silybum marianum* (SM) is a rich source of flavonolignans with promising antioxidant and antidiabetic properties; however, its therapeutic application is limited by poor stability and bioavailability. This study combined experimental and computational approaches to develop and evaluate SM-loaded chitosan nanoparticles (CS–SM nanoparticles). Microwave-assisted extraction followed by LC-MS/MS profiling identified eleven metabolites, including major flavonolignans characteristic of SM. Nanoparticles prepared by ionic gelation exhibited favorable physicochemical properties, including a particle size of 173–189 nm, a polydispersity index of 0.23, a zeta potential of +41.5 mV, an encapsulation efficiency of 98%, and a drug loading capacity of 50%, indicating the formation of a stable colloidal delivery system. CS–SM nanoparticles showed enhanced antioxidant, anti-inflammatory, and α-amylase inhibitory activities compared with crude extracts. The formulation exhibited an α-amylase IC_50_ value of approximately 0.40 mg/mL and maintained low hemolytic activity, suggesting favorable preliminary biocompatibility. Molecular docking demonstrated favorable interactions of neosilyhermin A, silibinin, and silyhermin with α-amylase and α-glucosidase active sites. Short-timescale molecular dynamics simulations revealed ligand-dependent behavior within the chitosan–TPP matrix, indicating different release tendencies among the investigated flavonolignans. Overall, the results support CS–SM nanoparticles as a promising platform for the delivery of bioactive phytochemicals with antioxidant and antidiabetic potential.

## 1. Introduction

Diabetes mellitus is a chronic metabolic disorder characterized by persistent hyperglycemia resulting from impaired insulin secretion, impaired insulin action, or both. Uncontrolled glycemia leads to severe long-term complications affecting the cardiovascular, renal, ocular and nervous systems, making diabetes one of the leading causes of global morbidity and mortality. According to the International Diabetes Federation, 589 million adults were living with diabetes in 2024, and this number is projected to rise to 853 million by 2050 [1]. More than 90% of these cases represent type 2 diabetes, driven by lifestyle, demographic, and environmental factors. Given this escalating burden, interest in alternative and complementary therapeutic strategies—particularly those based on plant-derived bioactive compounds—continues to grow [2].

Medicinal plants have been used for centuries and remain a valuable source of phytochemicals with broad therapeutic potential. They yield structurally diverse compounds with antioxidant, anti-inflammatory, antimicrobial, and metabolic effects, making them attractive candidates for drug discovery and supportive treatment approaches [3]. The Asteraceae family is particularly rich in phenolic acids, flavonoids, acetylenes, and terpenoids, which contribute to clinically relevant pharmacological activities, including antioxidant and anti-inflammatory effects [4].

One of its most biologically active members is *Silybum marianum* (SM), widely known as milk thistle. SM exhibits antioxidant, antidiabetic, antiproliferative, hepatoprotective, and antimicrobial properties [5]. Its main bioactive complex, silymarin, contains several flavonolignans such as silibinin, isosilybin, silychristin, and silydianin, with silibinin recognized as the most potent constituent [6]. These compounds have been associated with anticancer effects, reduction in chemotherapy-induced toxicity, and protection against UV-induced skin damage [7]. SM compounds exhibit anticancer activity, inhibit the proliferation of tumor cells, and prevent the hand-foot syndrome caused by capecitabin. They also lessen the adverse effects of chemotherapy [8]. This plant also lowers skin tumors and guards against UV-induced skin cancer when applied topically. Silymarin has also been utilized in individuals with breast cancer who have received chemotherapy or radiation therapy because of its chemo preventive properties [9].

Although SM has shown promise in diabetes management due to its active compound silymarin, conventional extracts suffer from poor solubility and limited bioavailability, restricting their therapeutic potential [10]. Nanotechnology offers a powerful solution, yet few studies have systematically developed chitosan-based nanoparticles to enhance the delivery of SM extract. Chitosan, a biocompatible and biodegradable polymer, provides controlled release and improved stability, but its application in SM-mediated diabetes therapy remains underexplored [11]. Moreover, while molecular modeling and molecular dynamics simulations can provide valuable insights into the interaction of silymarin with biomolecules and nanoparticle carriers, such computational approaches are rarely integrated with experimental studies [10]. Nanotechnology offers a promising opportunity to address these limitations. Chitosan—a biodegradable, biocompatible, and environmentally friendly polymer—forms nanoparticles (ChNPs) through ionic gelation with tripolyphosphate (TPP). Furthermore, chitosan has emerged as a wall material in microencapsulation due to its ability to form a cationic polymeric network, providing a protective layer surrounding the core. This enhances the efficiency of encapsulation, enhances the stability of storage, and ensures a controlled release of active ingredients including essential oils, pigments, and plant extracts [12,13,14].

However, relatively few studies have combined SM with chitosan-based nanocarriers for antidiabetic applications, and even fewer have integrated experimental characterization with molecular modeling. Existing literature lacks detailed mechanistic insight into how SM flavonolignans interact with key digestive enzymes such as α-amylase and α-glucosidase, or how these molecules behave dynamically within polymeric nanosystems [10,11]. This gap limits both the fundamental understanding and rational design of phytochemical-based nanomedicines.

Therefore, this study provides an eco-friendly and comprehensive approach by employing microwave-assisted extraction, which enhances the recovery of the polar flavonolignans from SM, thus enhancing the extraction efficiency while preserving the activity of the bioactive. Furthermore, the development of ionic gelation-based chitosan–TPP nanoparticle formulation along with molecular modeling provides an in-depth understanding of the molecular interactions and binding affinities of bioactives with α-amylase and α-glucosidase. Thus, the multidimensional incorporation of the green extraction and advanced nanocarrier design along with the in vitro and computational validation provides a novel therapeutic approach in type 2 diabetes management.

## 2. Results

### 2.1. Preparation of Plant Extract

Extraction of plant samples were performed at room temperature using a microwave assisted method. Aqueous, methanol, and ethanol were the three solvents used in experiments.

The highest percentage yield was observed in the ethanol extract. The percentage yield of the ethanol, methanol, and aqueous extracts was 20.7%, 3.71%, and 2.9%, respectively.

### 2.2. Preparation of Chitosan Based Nanoparticles

Chitosan nanoparticles (ChNP) were made by the ionic gelation method. The source of chitosan was crab shell having an off-white appearance in powder form and its pH ranged between 7.0–9.0 (molecular weight 10–20 KDa, CAS numeral 9012-76-4, and product code CB0660). The size of SM chitosan-based nanoparticles was 173.19 nm, shown by zeta sizer and positively charged with a 41.5 mV potential based on zeta potential (see Supplementary Materials File S1).

### 2.3. Instrumental Analysis

The prepared SM chitosan nanoparticles were subjected to comprehensive instrumental characterization to confirm successful encapsulation and evaluate their physicochemical properties.

#### 2.3.1. Fourier Transform Infrared Spectroscopy (FTIR)

**Figure 1:**
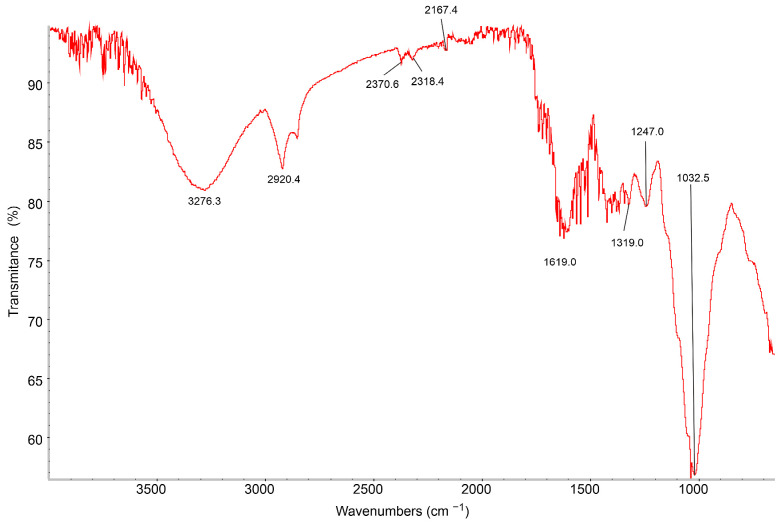
FTIR spectra of the SM plant extract.

The FTIR analysis confirmed the successful incorporation of the SM extract into the chitosan nanoparticle matrix. The spectrum (Figure 1) exhibited certain diagnostically significant absorption bands within the 600–3600 cm^−1^ region, each attributable to certain functional groups present in the formulation.

A wide and moderately intense absorption band was seen at 3278.3 cm^−1^ (T: 80.607%), which may be due to the overlapping O–H stretching vibrations from the phenolic hydroxyl groups present in the flavonolignan constituents of the SM extract, especially silybin, silydianin, and silychristin. At 2920.4 cm^−1^ (T: 82.665%), asymmetric C–H stretching vibrations of aliphatic methylene (–CH_2_–) and methine (–CH–) groups were observed, suggesting that the aliphatic structural framework of both chitosan and the SM extract was preserved during nanoparticle synthesis, confirming that the encapsulation process did not disturb the core chemical architecture of the components. Certain week bands were seen at 2370.6 cm^−1^ (T: 91.433%) and 2318.4 cm^−1^ (T: 91.933%), which reflects atmospheric CO_2_ intervention, cumulated double bond systems (C=C=O or C=C=C), or terminal alkyne C≡C stretching. Such low-intensity bands are usually considered as instrumental or environmental artifacts. The absorption band observed at 2167 cm^−1^ (T: ~91%) exhibited C=O stretching vibrations of conjugated carbonyl systems, a characteristic of the chromanone ring system predominantly present in the silybin and isosilybin. Thus, the presence of this band confirms the retention of the intact flavonolignan chromophoric system in nanoparticles, indicating the preserved pharmacophoric integrity of the SM constituents. Moreover, at the 1819.8–1341.2 cm^−1^ region, a cluster of absorption bands was observed, suggesting the characteristic interplay of the aromatic ring systems of silymarin with the polysaccharide framework of chitosan. The band at 1819.8 cm^−1^ (T: 79.784%) demonstrates a lactone or ester linkage due to C=O stretching. The bands at 1340.0 cm^−1^ (T: 79.876%) and 1341.2 cm^−1^ (T: 79.451%) are compatible with C–N stretching vibrations of - amine groups in chitosan, while O–H in-plane bending of the phenolic moieties is from the SM extract. The most important absorption bands observed in this FTIR spectrum occurred at wavenumbers of 1015.7 cm^−1^ (T: 56.692%) and 1032.5 cm^−1^ (T: 56.457%), which exhibited the highest transmittance loss in the whole spectrum. These intense absorptions represent the C-O-C stretching vibration bands of the glycosidic bonds in the backbone of chitosan and C-O stretching of the phenolic and secondary alcohol groups present in the flavonolignan structure. FTIR analysis of chitosan nanoparticles loaded with SM extract exhibited all the characteristic absorption peaks anticipated from both chitosan (N-H/O-H stretching, C-H aliphatic stretching, C-O-C glycosidic stretching) and flavonolignans of the silymarin class (phenolic O-H, conjugated C=O, aromatic C=C). Most importantly, the changes in peak positions and intensities, especially those in the 3278 cm^−1^ region (hydrogen bonding), 1341 cm^−1^ region (C-N/O-H bending vibration), and 1015–1032 cm^−1^ region (C-O stretching), suggest the presence of an intermolecular interaction between the SM extract and chitosan matrix, which is indicative of hydrogen bonding, electrostatic interactions between chitosan amino group protons and silymarin phenolic hydroxyl groups, and physical encapsulation within the core of the nanoparticles (Table 1).

#### 2.3.2. Scanning Electron Microscopy (SEM)

**Figure 2:**
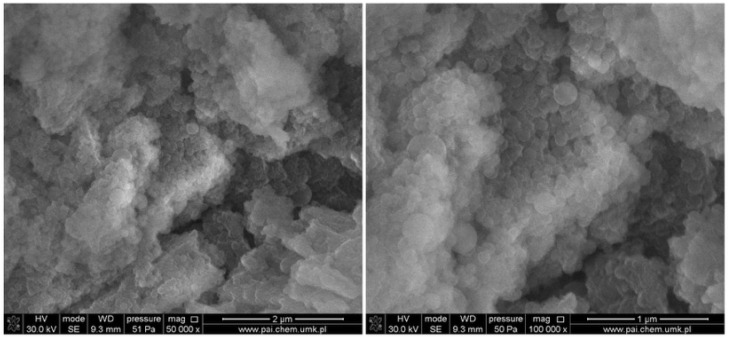
SEM micrographs of CS–SM nanoparticles at different magnifications.

Scanning electron microscopy (SEM) was used to investigate the morphology of the CS–SM nanoparticles. The micrographs presented in Figure 2 show the formation of predominantly spherical to quasi-spherical nanoparticles arranged in partially agglomerated clusters. The nanoparticle surfaces appear rough and heterogeneous, which is characteristic of chitosan-based systems prepared by ionic gelation. Similar morphologies have been reported for chitosan nanocarriers and other polysaccharide-based nanoparticle formulations [15,16].

The observed surface roughness may be associated with the incorporation of SM phytochemicals into the chitosan matrix. Such surface characteristics can increase the available surface area and may facilitate interactions with biological environments. The SEM images also reveal the presence of nanoparticle aggregates composed of smaller globular structures. Imaging was performed using an accelerating voltage of 10 kV and a working distance of 10.1 mm. Multiple magnifications were used to visualize both the overall morphology and individual nanoparticle features.

Furthermore, the SEM micrographs demonstrate partial clustering of globular nanoparticle structures, which is consistent with the hydrodynamic diameter of approximately 189.19 nm ±43.11 determined by dynamic light scattering (DLS). This size falls within the optimal range for nanocarrier systems and is typically associated with good dispersion behavior in aqueous media. The observed clustering can be attributed to the inherent properties of chitosan nanoparticles, as dehydration and vacuum conditions during SEM sample preparation often promote apparent aggregation in polysaccharide-based materials.

The morphology observed by SEM is consistent with the particle size results obtained by dynamic light scattering. The hydrodynamic diameter determined by DLS was 189.19 ± 43.11 nm, while SEM revealed nanoparticles within a comparable size range. The slightly larger size measured by DLS is expected because the hydrodynamic diameter includes the hydration layer surrounding the particles in suspension. Detailed DLS data are presented in Supplementary Materials File S1.

Partial agglomeration was observed in the SEM micrographs. This phenomenon is commonly reported for chitosan nanoparticles. It is frequently caused by dehydration and vacuum conditions during SEM sample preparation. Therefore, the clusters visible in Figure 2 do not necessarily indicate aggregation in an aqueous suspension.

The DLS results support this interpretation. The measured PDI value of 0.23 indicates acceptable size uniformity in suspension. In addition, the positive zeta potential of +41.5 mV indicates strong electrostatic stabilization of the nanoparticle system. These results suggest that the observed agglomeration is primarily a sample preparation artifact rather than evidence of poor colloidal stability. Detailed DLS and zeta potential data are provided in Supplementary Materials File S1.

Overall, the SEM analysis confirms the successful formation of CS–SM NPs. The observed morphology is consistent with the DLS and zeta potential measurements. Together, these results demonstrate that the developed nanoparticle system possesses appropriate structural integrity, nanoscale dimensions, and colloidal stability, supporting its potential application as a drug delivery carrier.

#### 2.3.3. Powder X-Ray Diffraction (PXRD) Analysis of SM–Chitosan Nanoparticles

The powder diffractograms were collected in the range of 5–50°2θ with an exposure time of 75 s/1° and step of 0.017° using a Phillips X’Pert Pro diffractometer equipped with an X’Celerator Scientific RTMS detector and equipped with an Empyrean Cu LFF X-ray tube (CuKα radiation).

**Figure 3:**
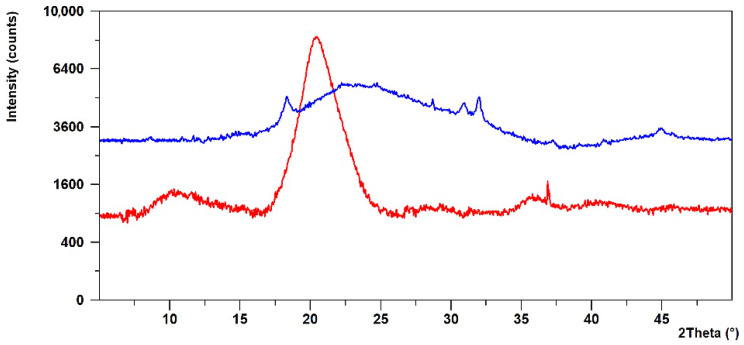
Powder diffractograms of chitosan (red) and CS–SM (blue).

Powder X-ray diffraction (PXRD) analysis provided additional insight into the structural organization of the nanoparticles. As shown in Figure 3, chitosan exhibited two broad halos at approximately 9.5° and 23° 2θ, reflecting its amorphous structure in agreement with earlier reports [17]. In contrast, the CS–SM displayed a sharp peak at 20–22° 2θ and several minor reflections in the 25–35° 2θ region, indicating the presence of crystalline phytochemicals such as flavonolignans. These crystalline signatures are consistent with previous PXRD analyses of S. marianum constituents [18,19]. The composite PXRD pattern demonstrates that encapsulation does not alter the amorphous nature of chitosan nor eliminate the crystalline signatures of the CS–SM, confirming that the flavonolignans are physically embedded within the chitosan matrix while maintaining structural compatibility.

#### 2.3.4. Zeta Size and Zeta Potential

**Figure 4:**
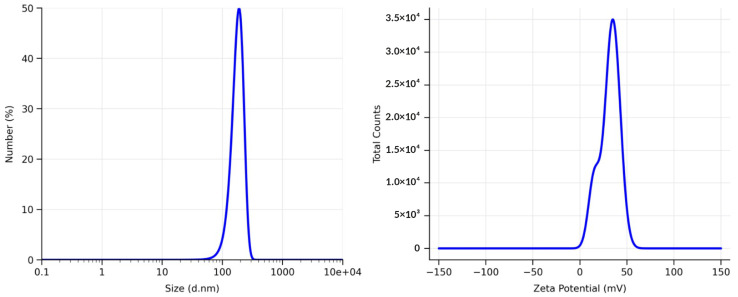
Particle size distribution of CS–SM nanoparticles obtained by DLS analysis (**left panel**) and zeta potential distribution profile (**right panel**).

Dynamic light scattering (DLS) analysis showed that the CS–SM nanoparticles formed a stable colloidal suspension with a hydrodynamic diameter of 189.19 ± 43.11 nm. This value is consistent with the particle size determined by zeta sizer analysis and SEM observations (~173.19 nm). The particle size distribution is presented in Figure 4 (left panel), while detailed measurement data are provided in Supplementary Materials File S1.

The zeta potential distribution is shown in Figure 4 (right panel). The nanoparticles exhibited a strongly positive surface charge of approximately +41.5 mV. Detailed zeta potential data are available in Supplementary Materials File S1. A zeta potential exceeding +30 mV is generally considered indicative of high colloidal stability. The obtained value suggests strong electrostatic repulsion between particles and a low tendency toward aggregation. The positive surface charge originates from protonated amino groups (–NH_3_^+^) of chitosan and may promote interactions with negatively charged biological membranes. UV–Vis spectroscopic analysis showed an absorption band near 189 nm, which can be attributed to electronic transitions associated with phenolic constituents present in CS–SM. The presence of this characteristic band supports the successful encapsulation of phytochemicals within the chitosan nanoparticle matrix. For more detailed data, see Supplementary Materials File S1.

Overall, the results presented in Figure 4 and Supplementary Materials File S1 confirm the successful formation of CS–SM nanoparticles. The combination of nanoscale particle size, acceptable PDI, and high positive zeta potential indicates a stable colloidal system with promising potential for drug delivery applications.

#### 2.3.5. UV–Visible Spectroscopy

UV–Vis spectroscopy was used to evaluate the incorporation of SM phytochemicals into the chitosan nanoparticles. The spectrum of the aqueous SM extract showed a pronounced absorption peak at approximately 250 nm. Additional absorption bands were observed between 288 and 310 nm. These bands are characteristic of flavonolignans such as silybin, silydianin, and silychristin. A gradual decrease in absorbance was observed near 325 nm. This feature is typical of flavonolignan-containing extracts. No significant absorption was detected above 450 nm. This observation suggests the absence of strongly absorbing impurities. Detailed spectra are presented in Supplementary Materials File S1.

The UV–Vis spectrum changed after nanoparticle formation. The characteristic bands of the free extract were reduced or disappeared. A dominant absorption peak appeared near 220 nm. This shift indicates interactions between the phytochemicals and the chitosan matrix. The observed hypsochromic shift suggests changes in the electronic environment of the encapsulated compounds. These changes may result from hydrogen bonding or electrostatic interactions. The spectral modifications support successful encapsulation of the extract in the nanoparticle matrix rather than simple physical mixing.

#### 2.3.6. % Encapsulation Efficiency (%EE)

The encapsulation efficiency was determined by measuring the amount of free extract remaining in the supernatant after centrifugation. The calculated %EE value was 98%. This result indicates that nearly all phytochemicals were incorporated into the nanoparticles. Only a small fraction remained unencapsulated. The low absorbance of the supernatant confirms this observation. Detailed results are presented in Table 2 and Supplementary Materials File S1.

The high %EE value demonstrates the strong affinity of SM phytochemicals for the chitosan matrix. Interactions between phenolic compounds and chitosan amino groups may contribute to this effect. High encapsulation efficiency is advantageous because it minimizes the loss of active compounds during nanoparticle preparation. It also increases the amount of bioactive material available for therapeutic action.

#### 2.3.7. Drug Loading % (DL%)

To measure the incorporation of the SM extract into nanoparticles, the drug loading (DL) was measured. A value of 75 mg of drug and 75 mg of chitosan was taken, and DL% was calculated as follows:

$$DL\%=\frac{Mass\;of\;drug\;loaded\;in\;nanoparticles\;(mg)\times 100}{Mass\;of\;drug\;loaded\;(mg)+Mass\;of\;chitosan\;(mg)}$$

$$DL\%=\frac{75\;mg\times 100}{75\;mg+75\;mg}$$

$$DL\%=\frac{75\;mg\times 100}{150\;mg}$$

DL%=50%

The drug loading percentage was used to evaluate the amount of extract incorporated into the nanoparticles. The calculated DL value was 50%. This means that half of the nanoparticle mass consisted of encapsulated SM extract. The remaining fraction corresponded to the chitosan carrier.

The obtained DL value indicates a high payload capacity, which is a desirable property in drug delivery systems. A high drug loading allows larger quantities of active ingredients to be delivered with a smaller amount of carrier material. The combination of 98% encapsulation efficiency and 50% drug loading demonstrates the effectiveness of the nanoparticle formulation process.

#### 2.3.8. IC_50_

The α-amylase inhibitory activity of CS–SM nanoparticles was further evaluated using a concentration–response assay. The results are presented in Table 3. A progressive increase in enzyme inhibition was observed with increasing nanoparticle concentration, demonstrating a clear dose-dependent response. Inhibition increased from 17.2% at the lowest tested concentration to 75.0% at the highest concentration. A 50% inhibition level was achieved at a concentration of 20, corresponding to an IC_50_ value of approximately 0.40 mg/mL under the applied experimental conditions.

The obtained concentration–response relationship confirms that the biological activity of the encapsulated phytochemicals was retained after nanoparticle formation. The relatively low standard deviation values observed across all measurements indicate good repeatability of the assay and support the reliability of the calculated IC_50_ value. These findings demonstrate that the CS–SM nanoparticle formulation possesses significant α-amylase inhibitory activity and may serve as a promising carrier system for antidiabetic phytochemicals.

#### 2.3.9. LC-MS/MS

LC-MS/MS analysis revealed a chemically diverse profile of metabolites across the aqueous, ethanolic, and methanolic extracts of SM (Table 4). A total of eleven compounds were identified, including major flavonolignans characteristic of SM—silibinin, silyhermin, neosilyhermin A, silydianin, silychristin and isosilybin isomers—as well as additional metabolites such as taxifolin, β-sitosterol, and dehydrodiconiferyl alcohol (see Supplementary Materials File S1).

It should be emphasized that the analysis was performed without chromatographic separation (column-free MS acquisition), and compound identification was based on mass-to-charge ratios and comparison with literature data. In line with this approach, the study was designed as a qualitative metabolite profiling, rather than quantitative or retention-based analysis.

Despite the absence of chromatographic separation, the detected metabolite profile remains consistent with previously reported LC-MS and chromatographic studies of SM extracts, supporting the reliability of the applied analytical strategy [18,20].

The ethanolic extract exhibited the most comprehensive chemical profile, containing nearly all detected flavonolignans and β-sitosterol. This observation aligns with existing evidence that ethanol effectively solubilizes the mid-polarity constituents of silymarin due to its amphiphilic properties [18]. The methanolic extract displayed a narrower yet more selective metabolite range, enriched in taxifolin and specific silyhermin derivatives—consistent with reports showing that methanol favors the extraction of low-molecular-weight, highly hydroxylated flavonoids [19].

The aqueous extract, although the least diverse chemically, retained hydrophilic metabolites such as silibinin and silyhermin, which corresponds with earlier hot-water extraction data [19]. β-Sitosterol was detected exclusively in the ethanolic fraction, reflecting its lipophilic nature—previous LC-MS/MS metabolomic profiling identified sterols only in solvents of moderate polarity [21].

### 2.4. Biological Test In Vitro Activities

#### 2.4.1. Antioxidant Activity

The antioxidant activity was evaluated using DPPH radical scavenging activity, total phenolic content (TPC), and total flavonoid content (TFC). The results are presented in Table 5.

As shown in Table 5, the aqueous extract exhibited the highest TPC (336.65 ± 0.42 mg GAE/mL) and TFC (264.08 ± 0.43 μg CE/mL) among the tested extracts. The aqueous extract also showed the strongest DPPH scavenging activity (48.10 ± 0.14%). These results indicate that water was the most effective solvent for the extraction of antioxidant phytochemicals from SM.

The methanolic extract exhibited intermediate TPC and TFC values but showed lower DPPH scavenging activity than the aqueous extract. The ethanolic extract exhibited the lowest TPC and TFC values and relatively low antioxidant activity. These findings suggest that phenolic and flavonoid compounds contributed substantially to the radical-scavenging properties of the extracts.

The CS–SM nanoparticles exhibited the highest antioxidant activity among all tested samples. As shown in Table 5, the nanoparticles reached a TPC value of 627.95 ± 0.09 mg GAE/mL and a TFC value of 553.99 ± 0.05 μg CE/mL. The nanoparticle formulation also showed the highest DPPH scavenging activity (66.47 ± 0.24%). All measured parameters were significantly higher than those observed for the free aqueous extract, as confirmed by Tukey’s HSD test.

One-way ANOVA followed by Tukey’s HSD test revealed significant differences among the investigated treatments (*p* << 0.05). The nanoparticle formulation exhibited significantly higher TPC, TFC, and DPPH scavenging activity than all crude extracts, as indicated by the distinct superscript letters in Table 5. The raw ANOVA output data are presented in Supplementary Materials File S1.

The enhanced antioxidant activity may be related to the efficient encapsulation of phytochemicals within the chitosan matrix. Chitosan can protect bioactive compounds against degradation and may contribute directly to antioxidant activity through its amino and hydroxyl groups. The high encapsulation efficiency obtained for the nanoparticles further supports this interpretation.

These results demonstrate that both solvent selection and nanoparticle encapsulation significantly influenced the antioxidant properties of the SM preparations.

Overall, the results presented in Table 5 demonstrate that nanoparticle encapsulation significantly enhanced the antioxidant potential of SM. The increases in TPC, TFC, and DPPH scavenging activity confirm the successful preservation and enrichment of antioxidant phytochemicals within the chitosan nanoparticle system.

#### 2.4.2. Antidiabetic Potential

##### Alpha Amylase Inhibition Assay

The antidiabetic potential of the tested samples was evaluated using an α-amylase inhibition assay. α-Amylase is a key enzyme involved in the initial stage of starch digestion. Inhibition of this enzyme can reduce postprandial glucose release. Therefore, α-amylase inhibition is commonly used as a preliminary indicator of antidiabetic activity [22]. Previous studies have demonstrated a significant α-glucosidase inhibitory activity of *Silybum marianum* extracts and their major phytochemical constituents [20,21,23,24,25]. However, the present study focused on α-amylase as a primary screening enzyme.

The quantitative results of α-amylase inhibition, hemolytic activity, and anti-inflammatory activity are presented in Table 6.

As shown in Table 6, the aqueous extract exhibited the highest α-amylase inhibitory activity among the tested extracts (78.84 ± 0.04%). The ethanolic extract showed moderate inhibitory activity (53.78 ± 0.04%), whereas the methanolic extract exhibited the lowest activity (42.32 ± 0.34%). These findings indicate that the extraction solvent strongly influenced the recovery of phytochemicals responsible for enzyme inhibition.

The observed trend was consistent with the antioxidant results. The aqueous extract exhibited the highest TPC and TFC values among the crude extracts. Phenolic compounds and flavonoids are recognized as important inhibitors of carbohydrate-hydrolyzing enzymes and may contribute to the observed antidiabetic activity [26,27,28]. Therefore, the superior α-amylase inhibition observed for the aqueous extract may be associated with its higher phytochemical content.

The CS–SM nanoparticles exhibited strong α-amylase inhibitory activity (74.05 ± 0.11%). Although slightly lower than that of the aqueous extract, the nanoparticle formulation showed significantly higher inhibitory activity than the ethanolic and methanolic extracts, as confirmed by Tukey’s HSD test. The enhanced activity suggests that nanoparticle encapsulation improved the biological performance of the extract.

Several factors may explain this effect. The nanoparticles exhibited an encapsulation efficiency of 98% and a drug loading capacity of 50%. These values indicate the efficient incorporation of phytochemicals into the chitosan matrix and enhanced retention of biologically active constituents. The formulation also exhibited a particle size of approximately 173–189 nm, a PDI value of 0.23, and a zeta potential of +41.5 mV. These parameters indicate a stable and well-dispersed colloidal system. Nanoparticles within this size range are considered suitable for drug-delivery applications and may improve interactions between active compounds and biological targets [29,30,31].

The concentration–response assay demonstrated a clear dose-dependent inhibition of α-amylase by the nanoparticle formulation. The inhibitory activity increased progressively with increasing concentration, reaching 75.0% at the highest tested concentration. The calculated IC_50_ value was approximately 0.40 mg/mL, confirming the ability of the encapsulated phytochemicals to retain their biological activity after nanoparticle preparation. The observed concentration-dependent response further supports the reliability of the measured inhibitory effect.

One-way ANOVA followed by Tukey’s HSD test revealed significant differences among the investigated treatments (*p* << 0.05). The distinct superscript letters shown in Table 6 indicate significant differences among the tested formulations. These results confirm that both extraction solvent and nanoparticle encapsulation significantly influenced α-amylase inhibitory activity. The raw ANOVA output data are presented in Supplementary Materials File S1.

Overall, the results presented in Table 6 demonstrate that nanoparticle encapsulation enhanced the antidiabetic potential of the SM extract. The high inhibition percentage, favorable IC_50_ value, high encapsulation efficiency, and excellent physicochemical properties support the potential application of CS–SM nanoparticles as carriers of antidiabetic phytochemicals. Similar improvements in biological activity after encapsulation have been reported for various chitosan-based nanoparticle systems containing plant-derived bioactive compounds [31,32,33].

##### Hemolytic Activity

Hemolytic activity was evaluated to assess the preliminary biocompatibility of *Silybum marianum* extracts and the CS–SM nanoparticle formulation. The results are presented in Table 6.

As shown in Table 6, all tested extracts and the nanoparticle formulation exhibited relatively low hemolytic activity compared with the positive control. The ethanolic extract showed the lowest hemolytic activity (2.10 ± 0.00%), followed by the methanolic extract (4.62 ± 0.11%) and the CS–SM nanoparticles (4.86 ± 0.06%). The aqueous extract exhibited the highest hemolytic activity among the investigated samples (5.34 ± 0.09%), although the observed value remained substantially lower than that of the positive control (94.57 ± 0.01%).

All samples exhibited hemolysis values below 10%, indicating low erythrocyte toxicity and acceptable preliminary biocompatibility of the tested formulations [34,35].

One-way ANOVA followed by Tukey’s HSD test revealed significant differences among the investigated treatments (*p* << 0.05). The distinct superscript letters shown in Table 6 indicate significant differences among individual formulations. These results confirm that the extraction solvent and nanoparticle formulation significantly influenced the hemolytic response.

The low hemolytic activity observed for the CS–SM nanoparticles indicates good compatibility with erythrocytes and suggests that nanoparticle encapsulation did not substantially increase membrane-disruptive effects. The obtained results are consistent with previous reports describing the favorable biocompatibility of chitosan-based delivery systems and SM-derived formulations [36].

Overall, the hemolysis assay confirmed the favorable biocompatibility profile of both the crude extracts and the nanoparticle formulation.

##### Anti-Inflammatory Activity

The anti-inflammatory activity of *Silybum marianum* extracts and CS–SM nanoparticles was evaluated using the bovine serum albumin (BSA) denaturation assay. The results are presented in Table 6.

As shown in Table 6, the CS–SM nanoparticles exhibited the highest anti-inflammatory activity (82.55 ± 0.02%) among all tested formulations. The ethanolic extract showed the strongest activity among the crude extracts (79.78 ± 0.01%), followed by the methanolic extract (72.36 ± 0.01%). The aqueous extract exhibited the lowest anti-inflammatory activity (64.44 ± 0.01%). These findings indicate that both the extraction solvent and nanoparticle formulation significantly influenced the anti-inflammatory properties of the SM preparations.

The enhanced anti-inflammatory activity observed for the nanoparticle formulation may be attributed to the efficient encapsulation and stabilization of bioactive phytochemicals within the chitosan matrix. Chitosan-based nanocarriers can improve the stability and availability of encapsulated compounds, thereby enhancing their biological activity. In addition, flavonolignans and phenolic constituents of *Silybum marianum* have been previously reported to possess anti-inflammatory properties through the inhibition of protein denaturation and modulation of inflammatory pathways.

One-way ANOVA followed by Tukey’s HSD test revealed significant differences among the investigated treatments (*p* << 0.05). The distinct superscript letters shown in Table 6 indicate significant differences among the tested formulations. These results confirm that both solvent selection and nanoparticle encapsulation significantly affected the anti-inflammatory activity.

The superior performance of the CS–SM nanoparticles suggests that nanoencapsulation enhanced the preservation and biological effectiveness of the active phytochemicals. The observed activity, together with the low hemolytic potential of the formulation, further supports the biocompatibility and therapeutic potential of the developed nanoparticle system.

Overall, the results presented in Table 6 demonstrate that nanoparticle encapsulation significantly improved the anti-inflammatory properties of the SM extract. These findings support the use of chitosan nanoparticles as an efficient delivery platform for anti-inflammatory phytochemicals derived from *Silybum marianum*.

### 2.5. Result of In Silico Study

#### 2.5.1. Molecular Docking

Docking simulations revealed distinct binding affinities and interaction patterns of the three SM flavonolignans toward both digestive enzymes: α-amylase (PDB ID: 1B2Y) and α-glucosidase (PDB ID: 5NN8). Table 7 summarizes the binding energies, predicted inhibition constants (pK_i_), and key hydrogen-bond interactions together with their geometric parameters within the active sites of the analyzed enzymes.

The combined analysis of binding energy, pK_i_, calculated inhibition constants (K_i_), and hydrogen-bond interactions provides a comprehensive understanding of ligand affinity toward α-amylase and α-glucosidase.

For α-amylase, neosilyhermin A exhibited the most favorable binding profile, characterized by the lowest binding energy (−9.24 kcal/mol), the highest pK_i_ value (6.77), and consequently the lowest K_i_ (~0.00016 mM; 0.16 µM). These parameters consistently indicate strong binding affinity. Structurally, this is supported by the formation of two hydrogen bonds with TYR62 and ILE235, exhibiting donor–acceptor distances of approximately 2.096–2.145 Å and favorable interaction energies (−1.51 and −1.89 kcal/mol). The agreement between energetic (E_B_), thermodynamic (pK_i_, K_i_), and structural (hydrogen bonding) descriptors confirms a stable and well-oriented ligand–enzyme complex.

Silibinin and silyhermin also demonstrated consistent trends across all evaluated parameters. Their binding energies (−8.44 and −8.45 kcal/mol) correspond to pK_i_ values of approximately 6.18–6.20 and K_i_ values in the submicromolar range (~0.00063–0.00079 mM). Importantly, these ligands interact with catalytically relevant residues such as TRP59, ASP197, and HIS201 (silibinin) and THR163 (silyhermin). In particular, the strong hydrogen bond formed by silyhermin (energy −4.096 kcal/mol, distance 1.982 Å) suggests a highly stabilizing interaction, which explains its favorable Ki despite a slightly weaker overall binding energy compared to neosilyhermin A.

In contrast, acarbose showed significantly weaker binding characteristics for α-amylase, with a higher binding energy (−6.55 kcal/mol), lower pK_i_ (4.80), and much higher K_i_ (~0.01585 mM; 15.85 µM). Although it forms hydrogen bonds with ASP197, ILE235, and HIS305, these interactions are characterized by relatively low interaction energies (−0.475 to approximately 0 kcal/mol), indicating weaker stabilization of the complex. Thus, both thermodynamic and structural descriptors consistently explain its lower inhibitory efficiency.

A similar relationship was observed for α-glucosidase. Neosilyhermin A again presented the most favorable combination of parameters (E_B_ = −8.32 kcal/mol, pK_i_ ≈ 6.1, K_i_ ≈ 0.00079 mM), supported by hydrogen bonding with ASP616. Silyhermin and silibinin exhibited intermediate profiles, with K_i_ values in the micromolar range (0.00251–0.00501 mM) and stabilizing interactions with catalytically important residues such as ASP282 and ASP616. The presence of multiple hydrogen bonds further compensates for slightly less favorable binding energies, emphasizing the importance of interaction networks in determining overall affinity.

Again, acarbose showed the weakest inhibition profile (E_B_ = −5.40 kcal/mol, pK_i_ ≈ 3.9, K_i_ ≈ 0.12589 mM), despite forming hydrogen bonds with ASP residues. Although geometrically acceptable, these interactions are moderate and insufficient to compensate for the weaker overall binding energy. Consequently, all evaluated descriptors consistently classified acarbose as the least potent inhibitor among the tested compounds.

Importantly, the integration of all parameters revealed a coherent pattern: lower binding energy corresponds to higher pK_i_, lower K_i_, and stronger inhibition, while strong and directional hydrogen bonds provide additional stabilization of the ligand–enzyme complex. Thus, convergence between global energetic descriptors, thermodynamic parameters, and structural features significantly strengthens the reliability of the observed trends.

However, the observed differences should be interpreted with caution. Docking scoring functions are inherently approximate and typically associated with an uncertainty of approximately 1–2 kcal/mol, which limits the reliability of fine ranking between compounds [34,35]. Therefore, although SM flavonolignans showed more favorable predicted binding energies than acarbose under the applied docking conditions, these differences should not be interpreted as definitive evidence of superior inhibitory potency. Instead, docking results primarily support the ability of these compounds to adopt stable binding conformations within enzyme active sites.

In particular, the presence of directional hydrogen bonds with catalytically relevant residues (e.g., ASP197, HIS201, ASP282, ASP616) and favorable geometric parameters suggests plausible interaction mechanisms consistent with previously reported studies on α-amylase and α-glucosidase inhibitors [36].

Overall, the docking analysis should be considered as providing qualitative structural insight rather than quantitative prediction of inhibitory strength.

**Figure 5:**
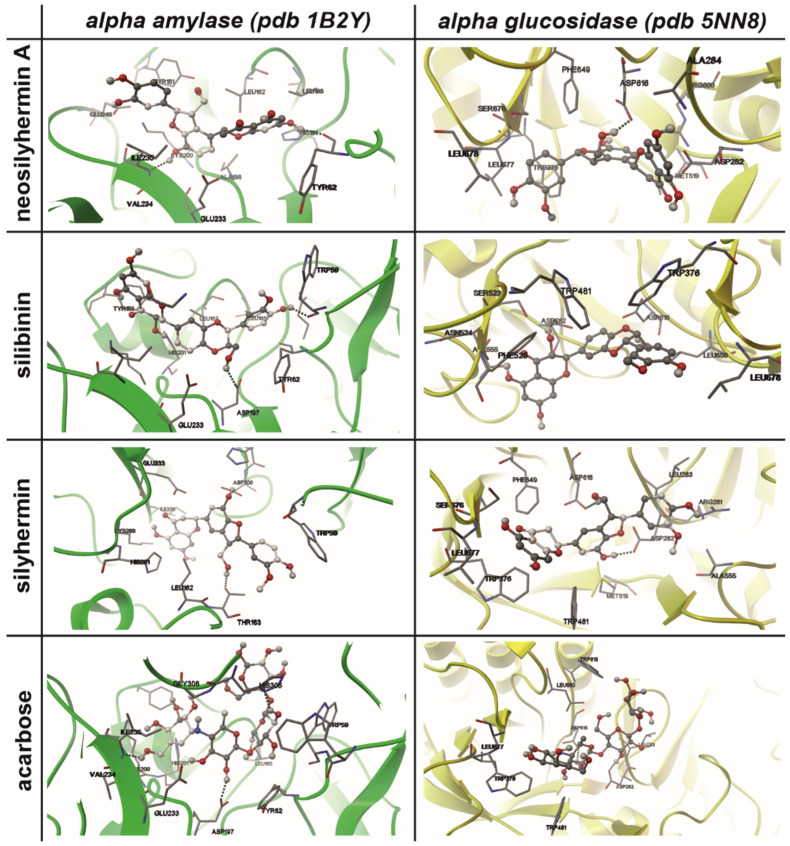
Binding modes of the investigated compounds.

Figure 5 illustrates the predicted binding poses of selected complexes, highlighting the consistency between the calculated energetic parameters and ligand orientation within enzyme active sites.

#### 2.5.2. Molecular Dynamics

Molecular dynamics simulations were performed using the NAMD simulation package [26]. Full-atom simulations were conducted with the CHARMM36 force field. Force-field parameters and charges were assigned to all molecules and ions present in the system. The TIP3P model was used to represent water molecules.

**Figure 6:**
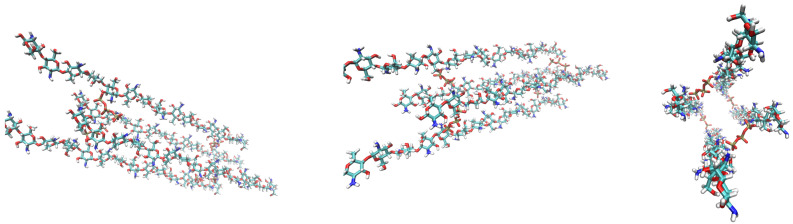
Graphical representation of the chitosan–TPP tubular carrier viewed from three different perspectives. The elongated structures correspond to chitosan chains, whereas the orange-red structures represent TPP anions.

The model consisted of a single chitosan–TPP tubular carrier containing one active molecule. Three systems were investigated independently. The active compounds included neosilyhermin A, silibinin, and silyhermin. The carrier structure was formed from four chitosan chains and four TPP anions. The arrangement of the chitosan chains and TPP molecules generated an internal cavity capable of accommodating one active compound molecule. The entire system was immersed in water. Twelve Na^+^ ions were added to ensure overall charge neutrality. Structure files for all molecular components, except water, were generated using CHARMM-GUI [27,28,29]. Initial molecular arrangements were prepared using Packmol [30,31]. Four chitosan chains and four TPP anions were organized into a tubular structure. One active molecule was subsequently inserted into the internal cavity. A distance tolerance of 2.0 Å was applied during system construction. This approach ensured that atoms belonging to different molecules were separated by at least 2.0 Å. Visual Molecular Dynamics (VMD) was used to visualize the molecular structures and prepare simulation systems (Figure 6) [32,33]. Water molecules and sodium ions were subsequently added. The resulting system was placed within a cuboid simulation box filled with water. After energy minimization, the longest dimension of the simulation box was approximately 189 Å and was oriented parallel to the *x*-axis. The remaining dimensions were approximately 123 Å and were oriented along the y- and z-axes.

The simulations were carried out in three stages. During the first stage, water molecules were minimized and equilibrated while the carrier structure was restrained. This procedure generated a properly equilibrated solvent environment. During the second stage, all components were released and allowed to move freely. The complete system was minimized and gradually heated from 0 K to 310 K. The third stage consisted of a production run conducted at 310 K and 1.01325 bar. Periodic boundary conditions were applied throughout the simulations. Langevin dynamics were used for temperature control. The integration time step was set to 2 fs.

The simulations were designed to evaluate the structural stability of the chitosan–TPP carrier and to investigate the release behavior of the encapsulated active compounds in an aqueous environment. Particular attention was paid to changes in the spatial relationship between the carrier and the active molecule during the simulation.

To quantify this process, the distance between the center of mass of the active compound and the center of mass of the carrier was calculated as a function of simulation time.

**Figure 7:**
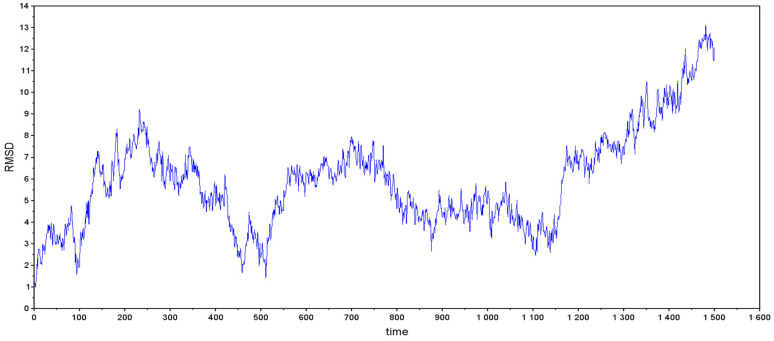
RMSD changes as a function of simulation time for the neosilyhermin A-loaded system.

A general trend was observed for all simulated systems. Progressive structural destabilization of the chitosan–TPP carrier occurred upon exposure to water. Therefore, RMSD analysis was employed to monitor changes in the relative position of the active compound with respect to the carrier throughout the simulation. The RMSD results for a three-nanosecond simulation obtained for neosilyhermin A are presented in Figure 7.

The system exhibited a progressive increase in RMSD over time. During the first two nanoseconds, moderate swelling of the carrier structure was observed. This process was followed by an increasing separation between the centers of mass of the carrier and the active compound. These observations indicate gradual disintegration of the carrier and progressive release of neosilyhermin A into the aqueous environment.

**Figure 8:**
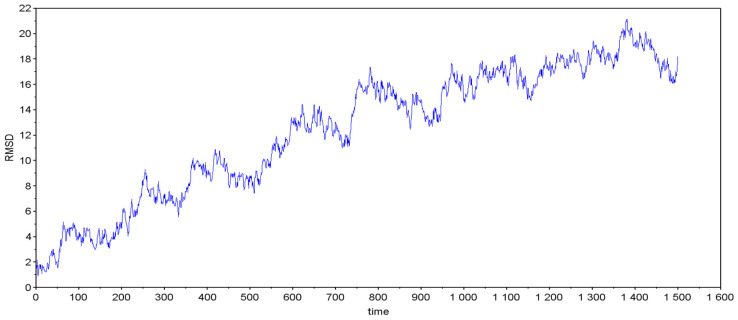
RMSD changes as a function of simulation time for the silibinin-loaded system.

The RMSD of a three-nanosecond simulation results for the silibinin-loaded system are shown in Figure 8. A continuous increase in the distance between the centers of mass was observed throughout the simulation. Progressive displacement of silibinin from the chitosan–TPP carrier was detected. Unlike the neosilyhermin A system, no pronounced swelling stage was identified. The results indicate gradual destabilization of the carrier structure and subsequent release of silibinin into the surrounding medium.

**Figure 9:**
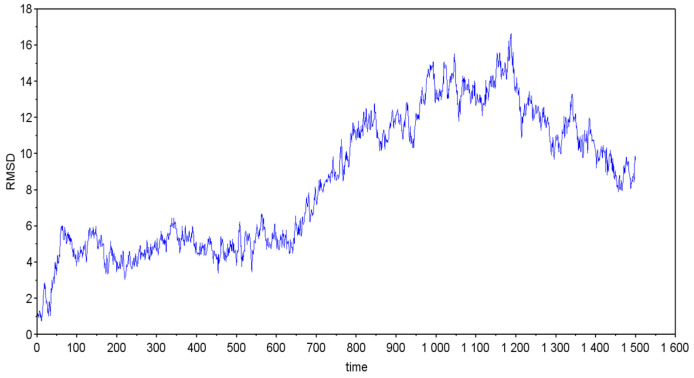
RMSD changes as a function of simulation time for the silyhermin-loaded system.

The RMSD profile of a three-nanosecond simulation obtained for the silyhermin-loaded system is presented in Figure 9.

The RMSD initially remained relatively stable and exhibited only moderate fluctuations. These fluctuations may correspond to hydration and swelling of the chitosan–TPP carrier. Subsequent separation between the centers of mass was observed, indicating structural destabilization of the carrier. At approximately 2 ns, a partial decrease in RMSD occurred (Figure 9). This behavior suggests the redistribution of carrier fragments around the active compound following disruption of the original tubular structure. Analysis of the simulation trajectory indicated that at later stages of the simulation, the active molecule remained associated with dispersed carrier fragments while occupying a larger region between the separated chitosan chains.

## 3. Discussion

The results obtained in this study demonstrate that both extracting solvent selection and nanoencapsulation significantly influence the physicochemical properties and biological performance of *Silybum marianum* (SM) preparations. Extraction yield and LC-MS/MS profiling revealed a clear relationship between solvent polarity and metabolite composition. The aqueous extract showed the highest recovery of hydrophilic bioactive constituents, whereas methanol favored the extraction of low-molecular-weight polyhydroxylated flavonoids. In contrast, ethanol produced the broadest metabolite profile, containing the majority of identified flavonolignans, which is consistent with the amphiphilic character of ethanol and its recognized suitability for the extraction of silymarin-derived compounds. These observations are in agreement with previously reported chromatographic and metabolomic studies of *S. marianum* extracts [18,19,20,21,37].

Physicochemical analyses confirmed the successful formation of CS–SM nanoparticles. FTIR spectroscopy demonstrated the presence of characteristic vibrational bands corresponding to hydroxyl, amine, and ether functionalities originating from both chitosan and SM phytochemicals. Changes in several absorption regions suggested hydrogen bonding and intermolecular interactions between flavonolignans and the polymer matrix. PXRD analysis showed that the chitosan carrier retained its predominantly amorphous character while preserving localized crystalline domains attributable to encapsulated phytochemicals. Similar structural characteristics have been reported for chitosan–TPP systems prepared using ionic gelation methods [17,38].

The SEM micrographs demonstrated the formation of spherical to quasi-spherical nanoparticles with partial aggregation. Such morphology is commonly reported for chitosan-based nanocarriers and is frequently attributed to dehydration during SEM sample preparation. Dynamic light scattering measurements supported these observations and revealed a hydrodynamic diameter of 189.19 ± 43.11 nm, a PDI value of 0.23, and a positive zeta potential of +41.5 mV. These parameters indicate good size homogeneity and high colloidal stability. Moreover, the strongly positive surface charge may promote electrostatic interactions with negatively charged biological membranes, potentially enhancing bioavailability and cellular uptake of the encapsulated phytochemicals [15,16].

The nanoparticle formulation exhibited exceptionally high encapsulation efficiency (98%) together with a drug loading capacity of 50%. These values suggest strong affinity between SM phytochemicals and the chitosan–TPP matrix. Efficient incorporation of flavonolignans into the carrier is particularly important because it minimizes the loss of active components during formulation and enables substantial phytochemical compound delivery while maintaining a relatively low amount of carrier material. The combination of high encapsulation efficiency and high loading capacity demonstrates the effectiveness of the ionic gelation approach used in this study.

The antioxidant evaluation demonstrated that nanoencapsulation substantially enhanced the measured biological activity of SM. CS–SM nanoparticles exhibited significantly higher total phenolic content, total flavonoid content, and DPPH scavenging capacity than the corresponding crude extracts. Such improvements may result from the protection of phenolic compounds against degradation, increased dispersibility of bioactive molecules, and stabilization provided by the chitosan matrix. Similar enhancements have previously been reported for chitosan-based systems containing flavonoid-rich plant extracts [15,39,40].

The antidiabetic evaluation revealed that the aqueous extract exhibited the highest α-amylase inhibitory activity among the tested formulations. This observation is consistent with the elevated TPC and TFC values measured for this extract and supports the widely recognized role of phenolic compounds and flavonoids as inhibitors of carbohydrate-hydrolyzing enzymes. Although slightly lower than the aqueous extract, the CS–SM nanoparticles maintained strong inhibitory activity and exhibited significantly higher inhibition than the ethanolic and methanolic extracts. This finding suggests that nanoencapsulation preserved the biological activity of the phytochemicals while providing additional formulation advantages such as enhanced stability and delivery potential [23,41,42].

The concentration-dependent inhibition profile further supported the antidiabetic potential of the nanoparticle system. The observed dose–response relationship yielded an IC_50_ value of approximately 0.40 mg/mL, confirming that the encapsulated phytochemicals retained their ability to inhibit α-amylase after nanoparticle preparation. The progressive increase in inhibitory activity with increasing concentration and the low experimental variability indicate good reproducibility and reliability of the assay.

The hemolysis assay demonstrated favorable preliminary biocompatibility of both crude extracts and nanoparticles. All tested formulations produced hemolysis values below 10%, indicating low erythrocyte toxicity. Furthermore, the hemolytic activity of the CS–SM nanoparticles remained relatively low despite the positive surface charge of the carrier. These observations are consistent with previous reports demonstrating good hemocompatibility of chitosan-based nanosystems and support the suitability of the developed formulation for further biological investigations [36].

The anti-inflammatory assay revealed that nanoparticle encapsulation further improved the biological activity of SM. The CS–SM nanoparticles exhibited the highest inhibition of protein denaturation among all tested formulations. The enhanced activity may result from the improved stabilization and availability of active flavonolignans within the chitosan matrix. These findings agree with previous reports demonstrating anti-inflammatory effects of both silymarin-derived metabolites and chitosan-based carriers [43,44].

The statistical analysis strongly supported the experimental observations. One-way ANOVA followed by Tukey’s HSD test revealed significant differences among treatments for all evaluated biological activities. The statistical results confirm that both solvent selection and nanoencapsulation significantly affected the antioxidant, antidiabetic, and anti-inflammatory properties of the investigated preparations.

Molecular docking studies provided complementary mechanistic insight into the experimentally observed antidiabetic activity. Among the investigated flavonolignans, neosilyhermin A consistently exhibited the most favorable binding parameters toward both α-amylase and α-glucosidase. However, the differences observed among the investigated compounds should be interpreted cautiously because docking scoring functions are associated with intrinsic uncertainties. Consequently, the docking results should primarily be regarded as qualitative evidence supporting the ability of SM flavonolignans to adopt favorable binding conformations within the enzyme active sites rather than as definitive predictors of inhibitory potency. Nevertheless, the identified interactions with catalytically important residues provide a plausible structural explanation for the observed enzyme inhibitory activity [34,35,36,45].

Because only short-timescale molecular dynamics simulations could be performed, the obtained results should be interpreted as preliminary mechanistic observations rather than definitive predictions of release behavior. The simulations revealed ligand-dependent differences in the early interaction of flavonolignans with the chitosan–TPP matrix. Neosilyhermin A and silibinin displayed progressive displacement from the carrier interior, whereas silyhermin showed a transient decrease in ligand–carrier distance associated with partial reassociation with carrier fragments. These findings indicate that molecular structure may influence the initial interaction pattern between flavonolignans and the polymer matrix. However, the 3-ns simulation timescale does not permit reliable conclusions regarding long-term release processes, carrier stability, or equilibrium properties [46,47,48].

Overall, the experimental and computational findings converge toward a coherent interpretation. Solvent polarity governs the phytochemical composition of SM extracts, while nanoencapsulation improves physicochemical stability and enhances several biologically relevant activities. The combination of high encapsulation efficiency, substantial drug loading, favorable colloidal properties, significant biological activity, and supportive molecular modeling results indicates that CS–SM nanoparticles represent a promising platform for the delivery of bioactive phytochemicals. These findings provide a foundation for future in vivo, pharmacokinetic, and controlled-release studies aimed at translating phytochemical-based nanomedicines into practical therapeutic applications.

## 4. Materials and Methods

### 4.1. Collection and Identification of Plant Material

Plant samples of SM were collected from a local retail market. Botanical authentication was performed at the Department of Botany, University of Agriculture, Faisalabad, Pakistan, where a voucher specimen (No. 37-1-24, dated 23 February 2024) was deposited for reference.

### 4.2. Preparatory Measures of SM Extracts

Extraction was performed at room temperature using a microwave-assisted method. Dried SM material (50 g) was mixed with 250 mL of solvent (aqueous, methanol, or ethanol; 1:5 ratio), ensuring consistency with subsequent LC-MS/MS analysis. Certain solvents (aqueous, methanol and ethanol) based on a wide polarity window were utilized. The sample was then heated in a microwave oven three times for 30 s to enhance the solvent penetration with minimum risk of the thermal degradation of bioactives. After cooling, mixtures were kept at room temperature for 24 h, filtered, and evaporated in a 52–55 °C water bath to obtain semi-solid extracts. Extracts were stored at 4 °C. These conditions were employed to enhance the extraction efficiency while maintaining bioactive stability and to ensure reproducibility across batches. For the biochemical assays, extracts were reconstituted in DMSO (1:4, *v*/*v*). Extracts with the highest in vitro activity were selected for nanoparticle preparation [49,50], and all solvent descriptions were standardized to match the analytical workflow used for LC-MS/MS.

Yield of plant extracts were calculated by the following formula:

$$Percentage\;Yield\;=\;\frac{Weight\;of\;obtained\;extract\;(g)\;\times 100}{Weight\;of\;plant\;sample\;(g)}$$

### 4.3. Characterization of SM Extracts

#### 4.3.1. FTIR Analysis

FTIR spectra were recorded using a Bruker Tensor 27 spectrometer. Samples were mixed with KBr, pressed into pellets, and scanned from 400 to 4000 cm^−1^ to identify functional groups [51].

#### 4.3.2. LC-MS/MS Analysis

Chemical profiles of the extracts were analyzed using a Shimadzu LCMS-8045 triple quadrupole system coupled with a Nexera X2 UHPLC. Aqueous, ethanolic, and methanolic extracts were evaluated, consistent with the corrected extraction protocol. Both positive and negative ESI modes were used. Data were processed with LabSolutions LCMS 5.86 software [52,53].

#### 4.3.3. UV–Visible Spectroscopy

UV-visible spectrophotometric analysis of the *Silybum marianum* extract and SM-loaded chitosan nanoparticles was conducted at the wavelength range of 200–800 nm.

### 4.4. Preparation of SM-Loaded Chitosan Nanoparticles (CS–SM Nanoparticles)

Nanoparticles were prepared via ionic gelation. Chitosan (3 mg/mL; DDA ≥ 75%) was dissolved in 0.5% acidified water and stirred at 900 rpm at room temperature (pH 3.6) until clear. Independently, the SM aqueous extract (75 mg) was dissolved in 2 mL DMSO and mixed with 10 mL tripolyphosphate (TPP; 1 mg/mL).

The TPP–extract mixture was added dropwise to the chitosan solution with continuous stirring at 45 °C for 2–3 h until a viscous dispersion formed. After centrifugation (15,000 rpm, 25 °C, 15 min), pellets were resuspended in distilled water and sonicated for 3 h to ensure homogeneity [15].

### 4.5. Encapsulation Efficiency (%EE)

Encapsulation efficiency was determined spectrophotometrically at 530 nm [15].

$$\%EE=\left(\frac{Total\;drug-Free\;drug\;in\;supernatant}{Total\;drug}\right)\times 100$$

### 4.6. Characterization of Nanoparticles

#### 4.6.1. Particle Size and PDI

Size distribution and polydispersity index were measured using DLS on a Beckman Coulter Delsa Nano C at 25 °C and a 90° angle. Samples were diluted (0.2 mL in 5 mL filtered water), and all measurements were performed in triplicate (*n* = 3) [54,55,56].

#### 4.6.2. SEM Morphological Analysis

Surface morphology was examined by SEM under a 10 kV accelerating voltage and 10.1 mm working distance. Multiple magnifications were used to assess nanoparticle shape and texture.

#### 4.6.3. XRD Analysis

PXRD patterns were collected using a Phillips X’Pert Pro diffractometer (Cu Kα radiation), scanning between 5–50° 2θ with 0.017° steps and 75 s exposure.

### 4.7. Antioxidant Evaluation

#### 4.7.1. Total Flavonoid Content (TFC)

**Figure 10:**
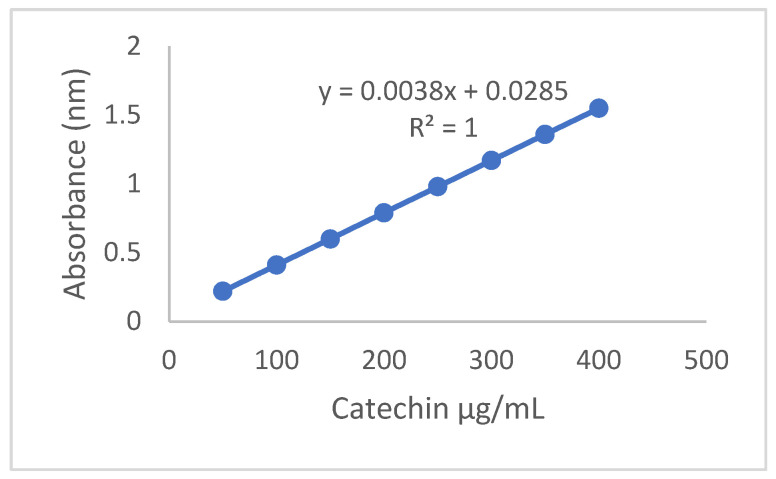
Graphical demonstration of a standard curve for TFC.

TFC was measured with the AlCl_3_ method at 415 nm and expressed as µg CE/mL The total flavonoid contents (TFCs) were given as µg CE/mL. The standard curve is shown in Figure 10 [57].

#### 4.7.2. Total Phenolic Content (TPC)

**Figure 11:**
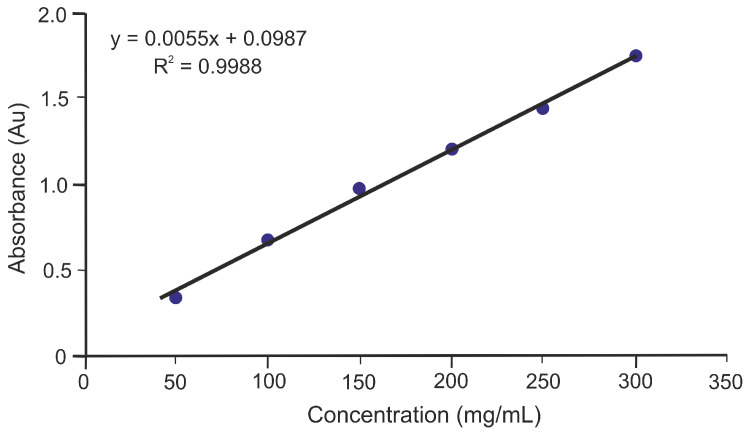
Graphical demonstration of a standard curve for TPC.

TPC was determined using Folin–Ciocalteu reagent at 765 nm and expressed as mg GAE/mL. A standard curve is shown in Figure 11 [58].

#### 4.7.3. DPPH Radical Scavenging Capacity (DPPH)

DPPH (0.004%) solution was mixed with the samples and incubated for 30 min in the dark. Absorbance was recorded at 570 nm, as optimized for the sample matrix; this wavelength was retained but now explicitly justified given the chromophoric interferences in SM extracts [59].

### 4.8. Antidiabetic Potential

#### Alpha-Amylase Inhibition Assay

Extracts and CS–SM nanoparticles (10 µL) were incubated with α-amylase (from *Bacillus subtilis*) followed by starch substrate. The reaction was stopped with HCl, complexed with iodine, and absorbance was measured at 570 nm [60].

Percentage inhibition of alpha-amylase was calculated by using the following formula:

$$\%\;inhibition\;=\;\frac{Absorbance\;of\;control\;-\;Absorbance\;of\;test\;sample\;\times \;100}{Absorbance\;of\;control}$$

### 4.9. Hemolytic Assay

Human erythrocytes from healthy individuals were procured from the Blood Bank at the University of Agriculture’s dispensary in Faisalabad. The blood was washed to isolate erythrocytes that were later incubated with samples for 30 min. After centrifugation, supernatants were measured at 576 nm. PBS and 0.1% Triton X-100 served as the negative and positive controls, respectively [44].

The following formula was applied to compute the percentage inhibition of hemolysis:

$$\%\;inhibition\;=\;\frac{Absorbance\;of\;sample\;-\;Absorbance\;of\;negative\;control\;\times \;100}{Absorbance\;of\;control}$$

### 4.10. In Silico Assay

#### 4.10.1. Molecular Docking

Molecular docking was performed to investigate the interaction mechanisms of the major SM flavonolignans—neosilyhermin A, silibinin and silyhermin—with two key carbohydrate-hydrolyzing enzymes. The crystal structures employed in this study were obtained from the RCSB Protein Data Bank and included human pancreatic α-amylase (PDB ID: 1B2Y), which contains a well-characterized (β/α)\_8_ TIM-barrel catalytic domain and essential Ca^2+^/Na^+^ coordination sites that contribute to structural stability and enzyme activity [61,62]. The second structure was human lysosomal acid α-glucosidase (PDB ID: 5NN8), featuring a deep active-site pocket defined by two conserved aspartate residues responsible for nucleophilic attack and acid–base catalysis during glycosidic bond hydrolysis [63]. Furthermore, the α-amylase employed in the in vitro study was procured from Bacillus subtilis due to its stability, easy production, and cost-effectiveness while human pancreatic alpha-amylase provides clinically-relevant simulations of physiological conditions. The use of both approaches allows for the practical benefits of microbial enzymes in laboratory assays along with their human homologs in computational analysis, providing another way to validate findings obtained by means of one of these methods. The fact that the structure of α-amylase is highly conserved across species, especially in its active center, further corroborates this approach.

Ligand structures for silibinin (ZINC02033589), silyhermin (ZINC14709118) and acarbose (ZINC85537042) were downloaded from the ZINC database, while the structure of neosilyhermin A (HMDB0030106) was retrieved from the Human Metabolome Database [64,65]. Prior to docking, all ligands underwent full geometry optimization using Gaussian 09 [66]. Geometry optimizations were carried out at the DFT/B3LYP level of theory with the 6-311+G(d,p) basis set in an aqueous environment simulated using the polarizable continuum model (PCM). Optimization convergence was achieved when the root-mean-square gradient dropped below 10^−6^ a.u., ensuring that each ligand reached a stable minimum on the potential energy surface. Optimized structures were exported via GaussView [67] and subsequently processed in AutoDockTools 1.5.7 by assigning Gasteiger partial charges, defining rotatable bonds, and saving the structures in PDBQT format [68].

Protein preparation was performed using AutoDockTools. Crystallographic ligands and non-essential water molecules were removed, polar hydrogens were added, and Kollman charges were assigned prior to conversion to PDBQT format [69]. Docking simulations were conducted using AutoDock 4.2 and the Lamarckian genetic algorithm. The grid box was centered on the catalytic residues of each enzyme, with dimensions set to 50 × 50 × 50 Å and a grid spacing of 0.375 Å to ensure full coverage of the active site. A total of 100 independent genetic algorithm runs was performed for each ligand, and resulting binding poses were clustered using a root-mean-square deviation threshold of 2.0 Å to identify the most representative conformations.

#### 4.10.2. Molecular Dynamics

Molecular dynamics (MD) simulations were performed to investigate the structural stability of the chitosan–TPP carrier systems and the behavior of encapsulated SM-derived phytochemicals in an aqueous environment. All simulations were carried out using the NAMD 3.0.1 software package [26]. Full-atom simulations were performed using the CHARMM36 force field. The TIP3P water model was applied to represent the solvent environment.

Three independent systems were constructed. Each system contained one active compound, namely neosilyhermin A, silibinin, or silyhermin. The carrier structure consisted of four chitosan chains and four TPP anions. Each chitosan chain contained 15 repeating units and carried a positive charge, whereas each TPP molecule carried five negative charges. The arrangement of the chitosan chains and TPP anions produced a tubular structure with an internal cavity capable of accommodating a single active compound molecule.

Structure files for all molecular components, except water, were generated using CHARMM-GUI [27,28,29]. Initial molecular arrangements were created using Packmol [30,31]. Four chitosan chains and four TPP anions were positioned to form the carrier structure, and one active compound molecule was placed within the internal cavity. A minimum intermolecular distance of 2.0 Å was imposed during system construction to prevent steric overlap between atoms belonging to different molecules. The generated structures were visualized and further processed using Visual Molecular Dynamics (VMD) [32,33].

The assembled carrier–ligand system was immersed in explicit water. Twelve Na^+^ ions were added to neutralize the total charge of the system. The solvated model was placed in a rectangular simulation box. After minimization, the dimensions of the simulation box were approximately 189 Å × 123 Å × 123 Å. The longest dimension was aligned parallel to the chitosan chains.

Prior to the production simulations, all systems were energy-minimized to eliminate unfavorable intermolecular contacts. Equilibration was performed in three stages. During the first stage, water molecules were equilibrated while the carrier structure remained restrained. During the second stage, all molecular components were released, and the entire system was gradually heated from 0 K to 310 K. Production simulations were subsequently performed at 310 K and 1.01325 bar. Periodic boundary conditions were applied in all directions. Temperature control was achieved using Langevin dynamics. A time step of 2 fs was used throughout the simulations.

The simulations were designed to evaluate the stability of the chitosan–TPP carrier and to investigate the release behavior of the encapsulated phytochemicals. Structural changes occurring during simulation were monitored using the distance between the center of mass (COM) of the active compound and the center of mass of the chitosan–TPP carrier.

The following descriptor was used:

$$RMSD\left(t\right)=|\vec{{r(t)}_{tube}^{cm}}-\vec{{r(t)}_{AC}^{cm}}|$$

where $\vec{{r(t)}_{tube}^{cm}}$ denotes the center-of-mass vector of the chitosan–TPP and $\vec{{r(t)}_{AC}^{cm}}$ denotes the center-of-mass vector of the active compound at time t.

This parameter was used to quantify the relative displacement of the active molecule with respect to the carrier during the simulation. Changes in RMSD were interpreted as indicators of carrier swelling, structural destabilization, and progressive release of the encapsulated compound. The calculated RMSD profiles were subsequently used to compare the dynamic behavior of neosilyhermin A, silibinin, and silyhermin within the chitosan–TPP carrier system.

### 4.11. Statistical Analysis

All measurements were performed in triplicate (*n* = 3), and data are presented as mean ± SD. Statistical analysis was carried out using one-way analysis of variance (ANOVA) under a completely randomized design (CRD). Tukey’s honestly significant difference (HSD) test was subsequently applied for multiple comparisons among treatment means. Statistical significance was accepted at *p* < 0.05. Means within the same column followed by different superscript letters were significantly different according to Tukey’s HSD test. The obtained F-values and *p*-values were used to evaluate the significance of treatment effects.

## 5. Conclusions

This study demonstrates that CS–SM nanoparticles represent a promising multifunctional delivery platform with enhanced biological activity compared with crude SM extracts. Differences in extraction yield and metabolite distribution across solvents confirmed the strong influence of solvent polarity on the phytochemical profile of *Silybum marianum*. LC-MS/MS profiling identified eleven major metabolites, including key flavonolignans, while FTIR, PXRD, SEM, UV–Vis, DLS, zeta potential, encapsulation efficiency, and drug-loading analyses confirmed the successful incorporation of SM phytochemicals into a chitosan–TPP nanoparticle system. The developed nanoparticles exhibited favorable physicochemical characteristics, including a particle size of approximately 173–189 nm, a PDI of 0.23, a zeta potential of +41.5 mV, an encapsulation efficiency of 98%, and a drug loading capacity of 50%. These parameters indicate efficient phytochemical incorporation, high colloidal stability, and suitability for drug delivery applications.

Encapsulation significantly enhanced antioxidant, α-amylase inhibitory, and anti-inflammatory activities relative to the corresponding crude extracts. The nanoparticle formulation also maintained low hemolytic activity, supporting its preliminary biocompatibility. Together, these findings demonstrate the beneficial effect of chitosan-based encapsulation on the functional performance of SM-derived phytochemicals.

Molecular docking provided qualitative mechanistic insight into the interactions of major SM flavonolignans with α-amylase and α-glucosidase. The results suggested favorable binding orientations and interactions with catalytically relevant residues, supporting a plausible structural basis for enzyme inhibition. However, these findings should be considered hypothesis-generating rather than definitive evidence of inhibitory superiority. Short-timescale molecular dynamics simulations further demonstrated ligand-dependent behavior within the chitosan–TPP matrix. Neosilyhermin A and silibinin exhibited progressive displacement from the carrier, whereas silyhermin showed partial redistribution and transient reassociation with carrier fragments. Although the simulations were limited to the early stages of system relaxation, the observed dynamic trends provide valuable mechanistic insight into ligand–carrier interactions and the initial behavior of encapsulated phytochemicals in an aqueous environment [70,71,72,73].

Overall, the experimental and computational findings support the potential application of CS–SM nanoparticles as carriers for bioactive phytochemicals with antioxidant and antidiabetic potential. The identified extraction–polarity–nanocarrier–dynamics relationships provide a useful framework for the future development of phytochemical-based nanosystems. Further in vivo, pharmacokinetic, and long-term release studies are required to validate the translational potential and therapeutic relevance of the proposed CS–SM nanoformulation.

## Figures and Tables

**Table 1:** FTIR results of SM for the identification of functional groups.

No.	Wavenumber (cm^−1^)	Compound Class	Functional Group	Intensity
1	3276.3	Phenols, flavonoids, amines	O–H stretching, N–H stretching	Medium
2	2920.4	Aliphatic chains	C–H stretching (CH_2_/CH_3_)	Medium–strong
3	2167.4	Overtone/combination band	Non-assigned (not ketenes/allenes)	Weak–medium
4	1340.0	Phenolics, polysaccharides	C–N stretching, O–H bending	Medium
5	1319.5	Phenolics, aromatic systems	Aromatic C–O, C–N stretching	Medium
6	1241.2	Flavonolignans, polyphenols, ethers	C–O stretching (aryl–O, C–O–C), C–N stretching	Medium–strong
7	1032.5	Polysaccharides, polyphenols	C–O stretching (alcohols, glycosides)	Strong
8	1015.7	Carbohydrates, phenolic ethers	C–O stretching	Strong
9	920.7	Aromatic systems	C–H out-of-plane bending	Medium
10	910.9	Aromatic systems	C–H out-of-plane bending	Medium
11	820.7	Aromatic rings	C–H bending (1,2,4-substituted rings)	Strong
12	800.8	Aromatic ring deformation	C–H bending	Strong
13	790.9	Aromatic vibration	C–H deformation	Strong
14	560.5	Polysaccharide fingerprint region	Skeletal carbohydrate vibrations	Medium

**Table 2:** % encapsulation efficiency (%EE) of the CS–SM nanoparticles.

Sr. No.	Absorbance (530)	
	Supernatant	Extract
1	0.1082	3.496
2	0.1004	3.498
3	0.1098	3.489
Total	0.3184	10.483
Mean	0.106133	3.494333
N	3	3
SD	0.004106	0.003859
SEM	0.002371	0.002228
R^2^	0.9838	
%EE	98%	

**Table 3:** IC_50_ value of the CS–SM nanoparticles.

Concentration	Absorbance (Mean ± SD, *n* = 3)	Inhibition (%) (Mean ± SD)
0	0.824 ± 0.002	0.0 ± 0.0
5	0.682 ± 0.015	17.2 ± 0.015
10	0.538 ± 0.012	34.7 ± 0.012
20	0.412 ± 0.010	50.0 ± 0.010
30	0.329 ± 0.009	60.0 ± 0.009
40	0.247 ± 0.011	70.0 ± 0.011
50	0.206 ± 0.008	75.0 ± 0.008

**Table 4:** Identified phytochemical constituents in SM extracts based on LC-MS/MS analysis.

Compound	MW (g/mol)	Ion (+)	Ion (–)	Aqueous	Ethanol	Methanol
Neosilyhermin A	466.40	467.4	465.4	−	+	+
Silibinin	482.40	483.4	481.4	+	+	−
Silyhermin	466.40	467.4	465.4	+	−	+
Isosilybin A	482.40	483.4	481.4	−	+	−
Isosilybin B	482.40	483.4	481.4	−	+	−
β-Sitosterol	414.70	415.7	413.7	−	+	−
Taxifolin	304.25	305.25	303.3	−	−	+
Silychristin	482.40	483.4	481.4	−	+	−
Silydianin	482.40	483.4	481.4	−	+	−
Dehydrodiconiferyl alcohol	358.40	359.4	357.4	−	+	−

**Table 5:** Antioxidant activity of *Silybum marianum* extracts and CS–SM nanoparticles.

Plant Extract	TPC (mg GAE/mL)	TFC (μg CE/mL)	DPPH Scavenging (%)
Ethanol	147.82 ± 0.32 d	100.57 ± 0.33 d	39.42 ± 0.14 c
Methanol	293.71 ± 0.32 c	171.97 ± 0.43 c	30.94 ± 0.14 d
Aqueous	336.65 ± 0.42 b	264.08 ± 0.43 b	48.10 ± 0.14 b
Aqueous SM nanoparticles	627.95 ± 0.09 a	553.99 ± 0.05 a	66.47 ± 0.24 a

Values are expressed as mean ± SD (*n* = 3). Different letters within the same column indicate significant differences according to Tukey’s HSD test (*p* << 0.05).

**Table 6:** Biological activities of *Silybum marianum* extracts and CS–SM nanoparticles.

Plant Extract	α-Amylase Inhibition (%)	Hemolytic Activity (%)	Anti-Inflammatory Activity (%)
Ethanol	53.78 ± 0.04 c	2.10 ± 0.00 c	79.78 ± 0.01 b
Methanol	42.32 ± 0.34 d	4.62 ± 0.11 b	72.36 ± 0.01 c
Aqueous	78.84 ± 0.04 a	5.34 ± 0.09 a	64.44 ± 0.01 d
Aqueous SM nanoparticles	74.05 ± 0.11 b	4.86 ± 0.06 b	82.55 ± 0.02 a
Positive control	97.55 ± 0.42	94.57 ± 0.01	86.40 ± 0.01

Values are expressed as mean ± SD (*n* = 3). Different letters within the same column indicate significant differences according to Tukey’s HSD test (*p* << 0.05).

**Table 7:** Summary of the α-amylase and α-glucosidase docking experiment results.

Complex		E_B_ [kcal/mol]	pK_i_	Key Interactions	L_HB_ [Å]	E_HB_ [kcal/mol]
Protein	Ligand					
α-Amylase (pdb 1B2Y)	Neosilyhermin A	−9.24	6.77	TYR62	2.145	−1.508
				ILE235	2.096	−1.893
	Silibinin	−8.44	6.18	TRP59	1.930	−2.004
				ASP197	2.034	−3.072
				HIS201	2.008	−0.075
	Silyhermin	−8.45	6.20	THR163	1.982	−4.096
	Acarbose	−6.55	4.80	ASP197	1.998	−0.475
				ILE235	1.891	−0.299
				HIS305	1.968	−0.001
α-Glucosidase (pdb 5NN8)	Neosilyhermin A	−8.32	6.10	ASP616	1.899	−0.305
	Silibinin	−7.20	5.28	ASP282	1.807	−2.735
	Silyhermin	−7.60	5.57	ASP282	1.908	−2.711
				ASP616	2.084	−0.731
	Acarbose	−5.40	3.96	ASP282	1.779	−2.209
				ASP616	1.865	−1.553

## Data Availability

All data are included in the manuscript.

## Supplementary Materials

The following supporting information can be downloaded at: https://www.mdpi.com/article/10.3390/molecules31152677/s1; Supplementary Materials File S1.
