# Supplementary material for: Eco-Friendly Synthesis, Physicochemical Characterization, and In Vitro Biological Evaluation of Plant-Derived Bioactive-Loaded Chitosan Nanoparticles Supported by Molecular Modeling Studies
Source: Molecules. 2026 Jul 31;31(15):2677. doi: 10.3390/molecules31152677 (PMC13468664; doi:10.3390/molecules31152677)
Supplement: Supplementary file 1 [file molecules-31-02677-s001.zip › molecules-4205740-supplementary file S1.pdf]

Anton Paar

Measurement information

**Measurement name**	Untitled 28	**User**	AntonPaar
**Method**	-	**Time**	5/12/2024 11:13:44 PM
**Status**	Succeeded	**Instrument type**	Litesizer 500
**Measurement mode**	Particle size		
**Sample ID**	-		
**Batch number**	-		
		**Filter optical density**	0.553 (Automatic)
**Measurement cell**	Quartz	**Focus position**	0.0 mm (Automatic)
**Measurement angle**	Back scatter (Automatic)	**Material**	Unknown material
**Target temperature**	25.0 °C	**Material refractive index**	-
**Equilibration time**	0h 01m 00s	**Material absorption index**	-
**Analysis model**	Narrow	**Solvent**	Water
**Cumulant model**	Advanced	**Solvent refractive index**	1.3303
**Processed runs**	3 (quick)	**Solvent viscosity**	0.0008903 Pa.s
**Time for each run**	0h 00m 10s (quick)		

Particle size distribution (intensity)

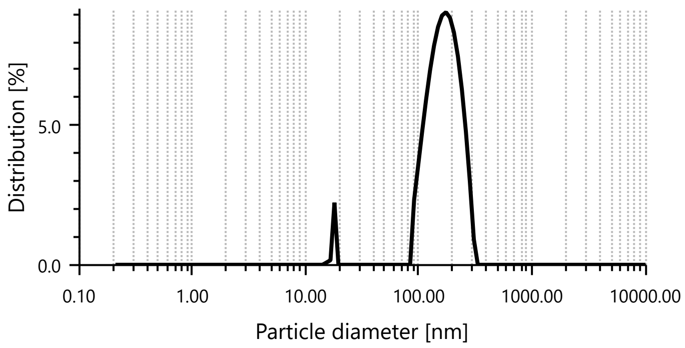

Result

**Hydrodynamic diameter**	189.01 nm	**Mean intensity**	197.7 kcounts/s
**Polydispersity index**	19.2 %	**Absolute intensity**	705.6 kcounts/s
**Diffusion coefficient**	2.6 µm²/s	**Intercept g1²**	0.3009
**Transmittance**	60.6 %	**Baseline**	1.009

Particle size distribution peaks (intensity)

Peak name	Size [nm]	Area [%]	Standard deviation [nm]
Peak 1	173.34	97.60	52.52
Peak 2	17.93	2.40	0.37
Peak 3	-	-	-

Anton Paar

Measurement information

**Measurement name**	Untitled 5	**User**	AntonPaar
**Method**	-	**Time**	5/3/2024 3:42:34 AM
**Status**	Succeeded	**Instrument type**	Litesizer 500
**Measurement mode**	Zeta potential		
**Sample ID**	-		
**Batch number**	-		
		**Filter optical density**	2.1731
**Measurement cell**	Omega cuvette Mat.No. 225288	**Solvent**	Water
**Target temperature**	25.0 °C	**Solvent refractive index**	1.3303
**Equilibration time**	0h 01m 00s	**Solvent viscosity**	0.0008903 Pa.s
**Henry factor**	1.5 (Smoluchowski)	**Solvent relative permittivity**	78.37
**Adjusted voltage**	200.0 V (Automatic Mode)		
**Processed runs**	100 (Automatic)		

Zeta potential distribution

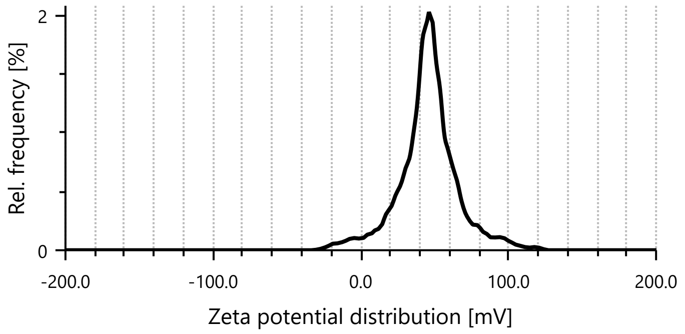

Result

**Mean zeta potential**	41.5 mV	**Mean intensity**	492.1 kcounts/s
**Standard deviation**	0.8 mV	**Filter optical density**	2.1731
**Distribution peak**	46.8 mV	**Conductivity**	0.143 mS/cm
**Electrophoretic Mobility**	3.2341 µm*cm/Vs	**Transmittance**	50.3 %

UV visible spectrum of Aqueous extract of Silybum marianum extract

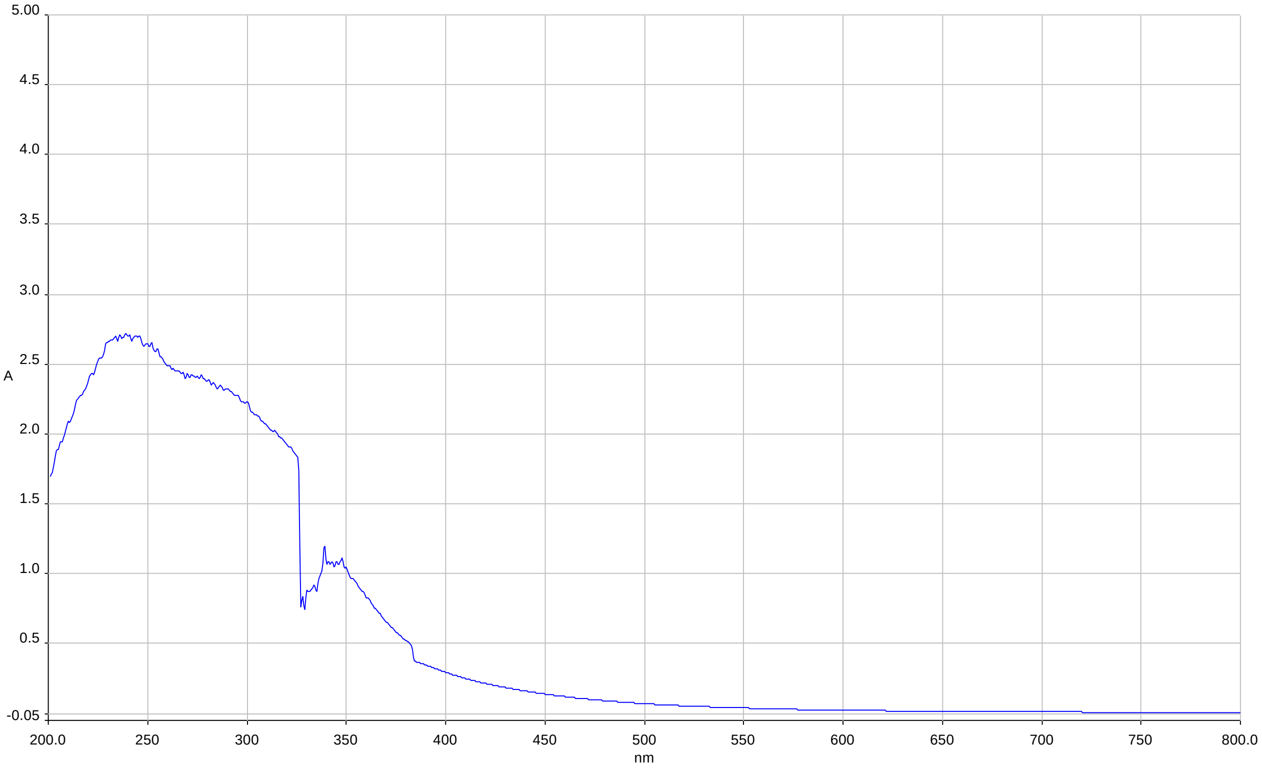

Silybum marianum loaded chitosan nanoparticles

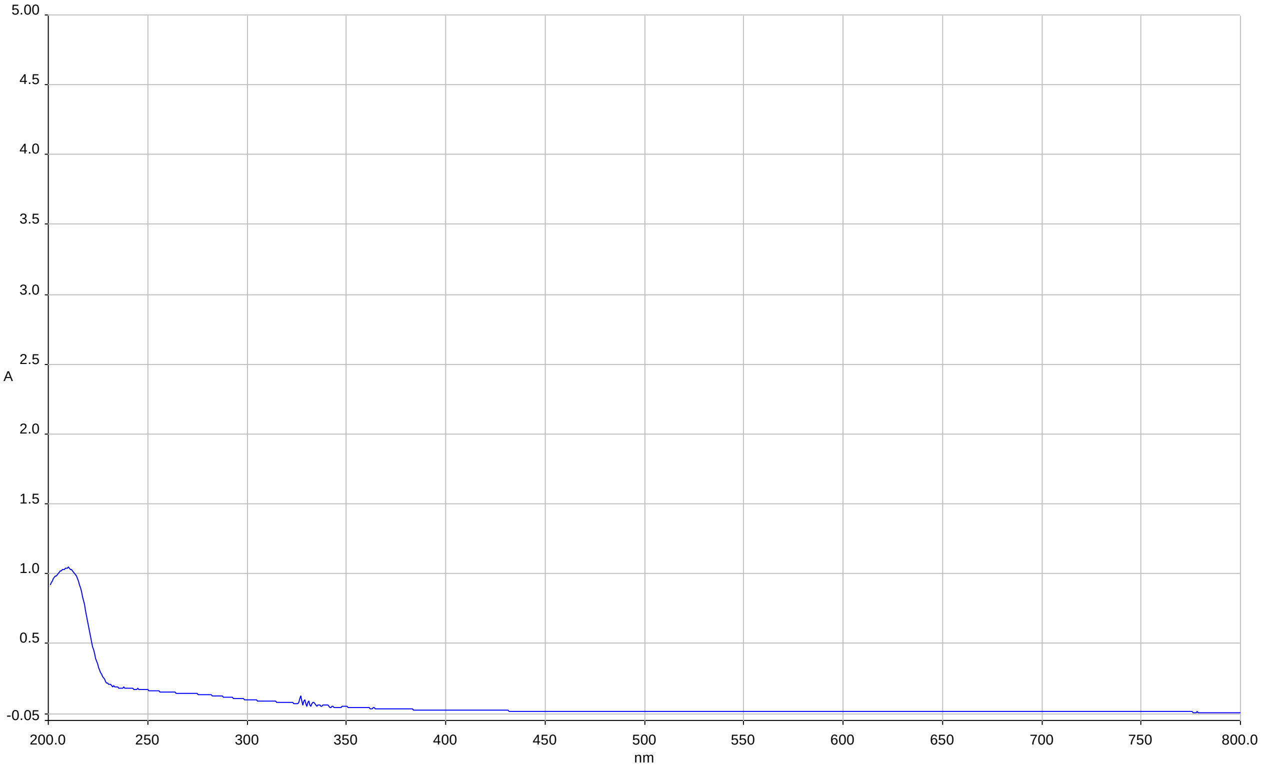

Table S1. Comparative analysis of Antioxidant activities among different treatments (such as DPPH= 2,2 Diphenyl-1-picrylhydrazyl; TPC= Total Phenolic content; TFC= Total Flavonoid content) with significance level α = 0.05 and p-value < $1x10^{-4}$

**DPPH**				
**Source**	DF	Adj SS	Adj MS	F-Value
**Factor**	3	1876.87	625.622	95.2
**Error**	8	52.59	6.574	
**Total**	11	1929.45		
**TPC**				
**Source**	DF	Adj SS	Adj MS	F-Value
**Factor**	3	138263	46087.72	21989
**Error**	8	17	2.1	
**Total**	11	138280		
**TFC**				
**Source**	DF	Adj SS	Adj MS	F-Value
**Factor**	3	98095.5	32698.5	13281
**Error**	8	19.7	2.5	
**Total**	11	98115.2		

DF: Degrees of Freedom; Adj SS: Adjusted Sum of Squares; Adj MS: Adjusted Mean Square; F-value Fisher's F statistic; p-value: Probability value; DF: degree of freedom.

Table S2. Comparative (completely randomized ANOVA) for Antidiabetic, Hemolytic and Anti-inflammatory activities among different treatments with significance level α = 0.05 and p-value < $1x10^{-4}$

Completely Randomized ANOVA for Anti-diabetic activity				
Source	DF	Adj SS	Adj MS	F-Value
Factor	3	1867.3	622.433	629
Error	8	7.92	0.990	
Total	11	1875.22		
Completely Randomized ANOVA for Hemolytic Activity				
Source	DF	Adj SS	Adj MS	F-Value
Factor	3	122.636	40.8788	44.3
Error	8	7.375	0.9219	
Total	11	130.001		
Completely Randomized ANOVA for Anti-Inflammatory Activity				
Source	DF	Adj SS	Adj MS	F-Value
Factor	3	1473.14	491.045	492
Error	8	7.98	0.998	
Total	11	1481.12		

DF: Degrees of Freedom; Adj SS: Adjusted Sum of Squares; Adj MS: Adjusted Mean Square; F-value: Fisher's F statistic; p-value: Probability value.

1. LC-MS/MS data: Aqueous extract of *Silybum marianum*

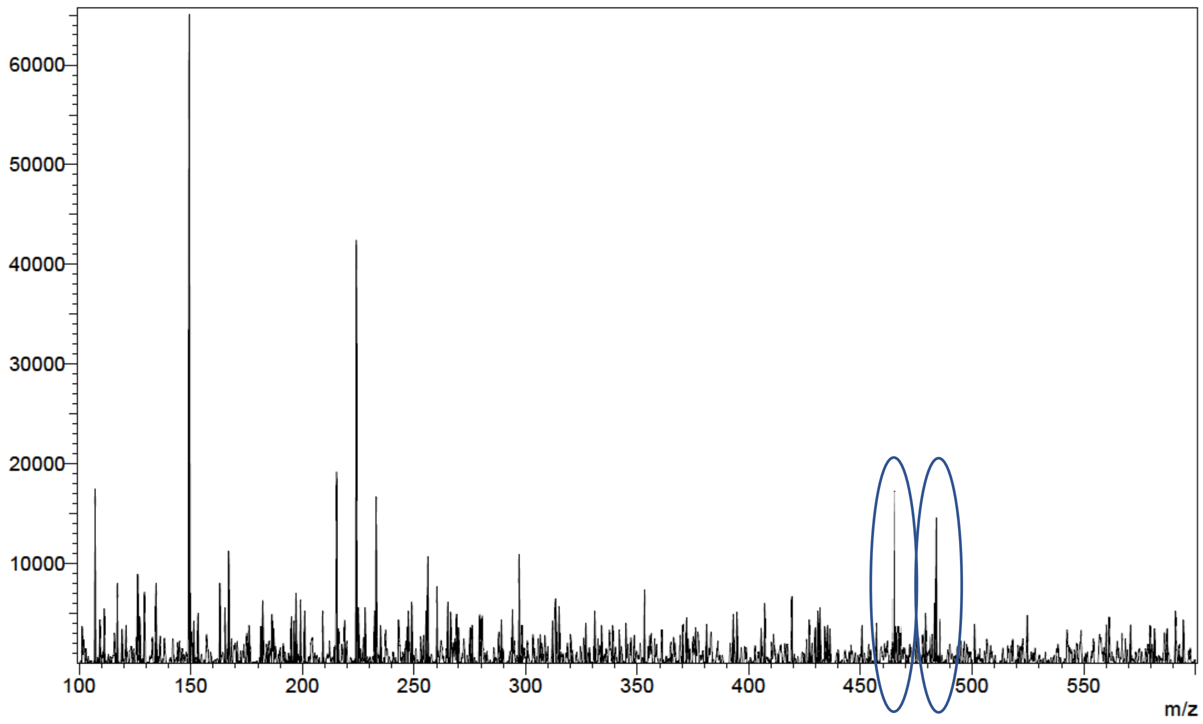

Figure S1 MS spectrum of aqueous extract

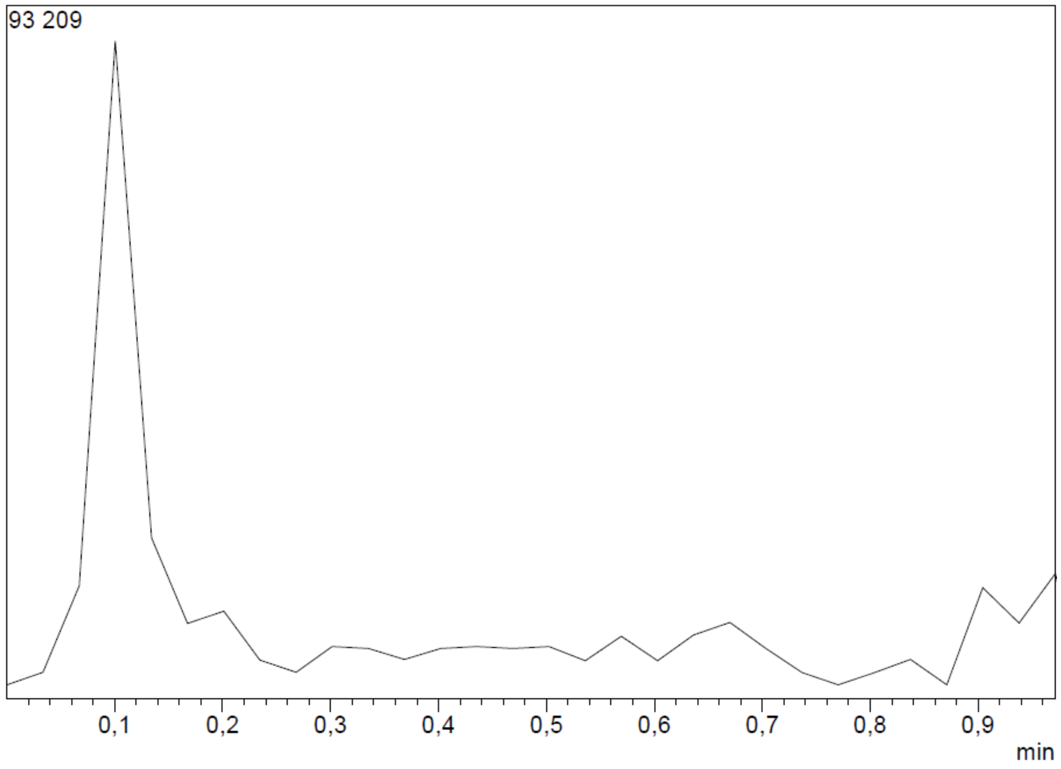

Figure S2 Chromatogram of Silibinin (m/z = 482.40) of aqueous extract

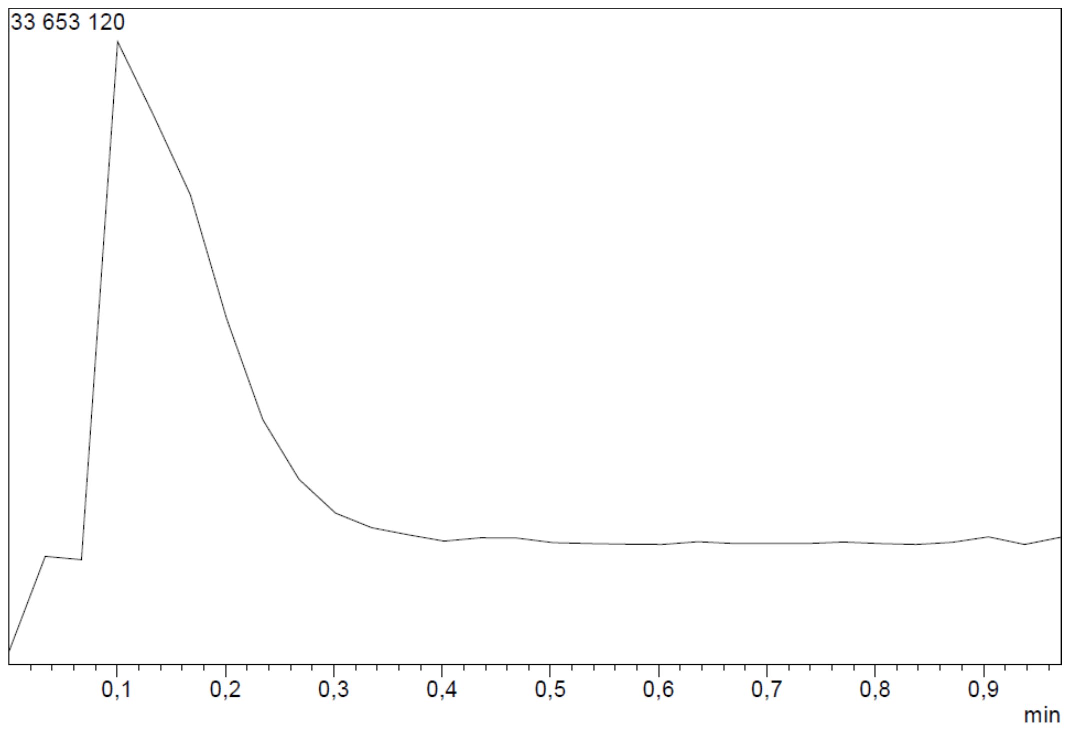

Figure S3 Chromatogram of Silyhermin (m/z = 466.40) of aqueous extract

2. LC-MS/MS data: Ethanolic extract of *Silybum marianum*

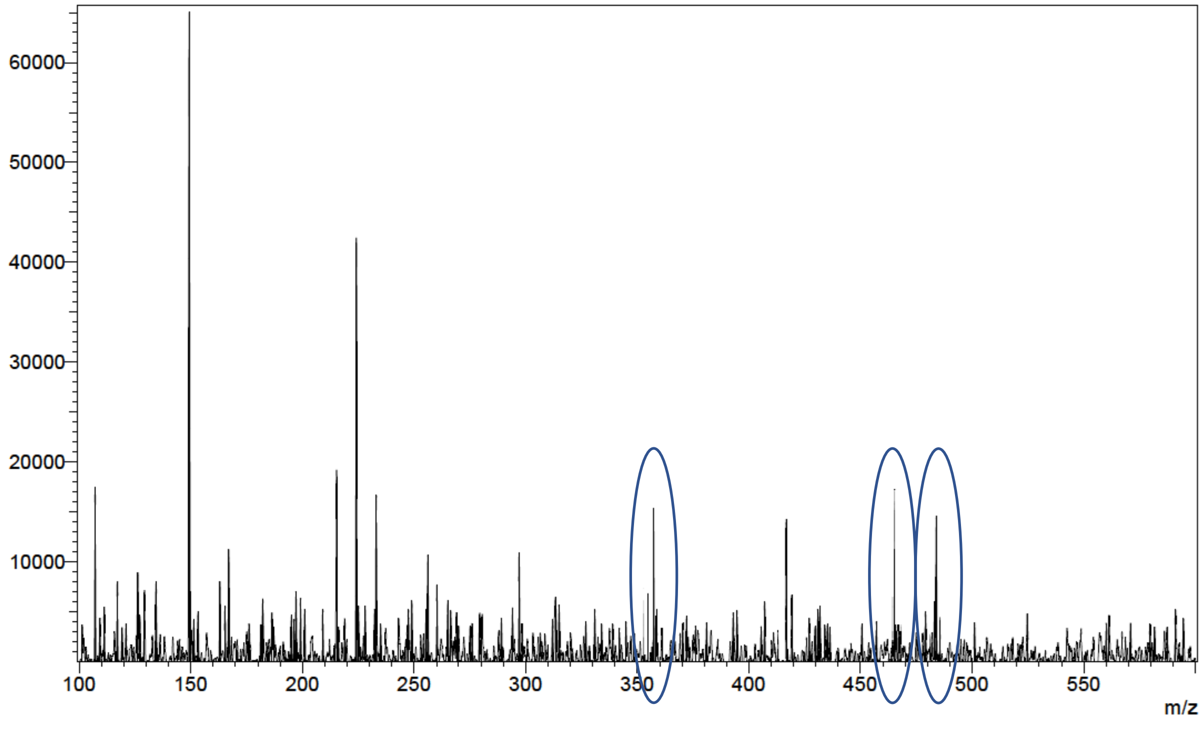

Figure S4 MS spectrum of ethanolic extract

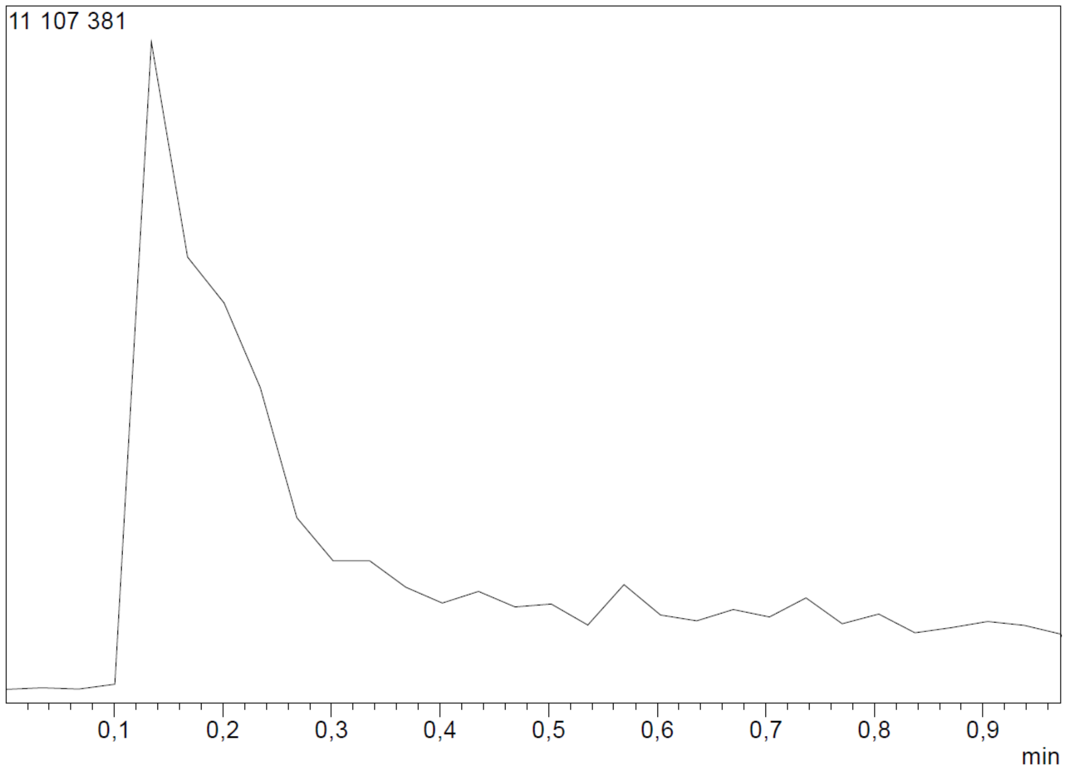

Figure S5 Chromatogram of Neosilyhermin A (m/z = 466.40) of ethanolic extract

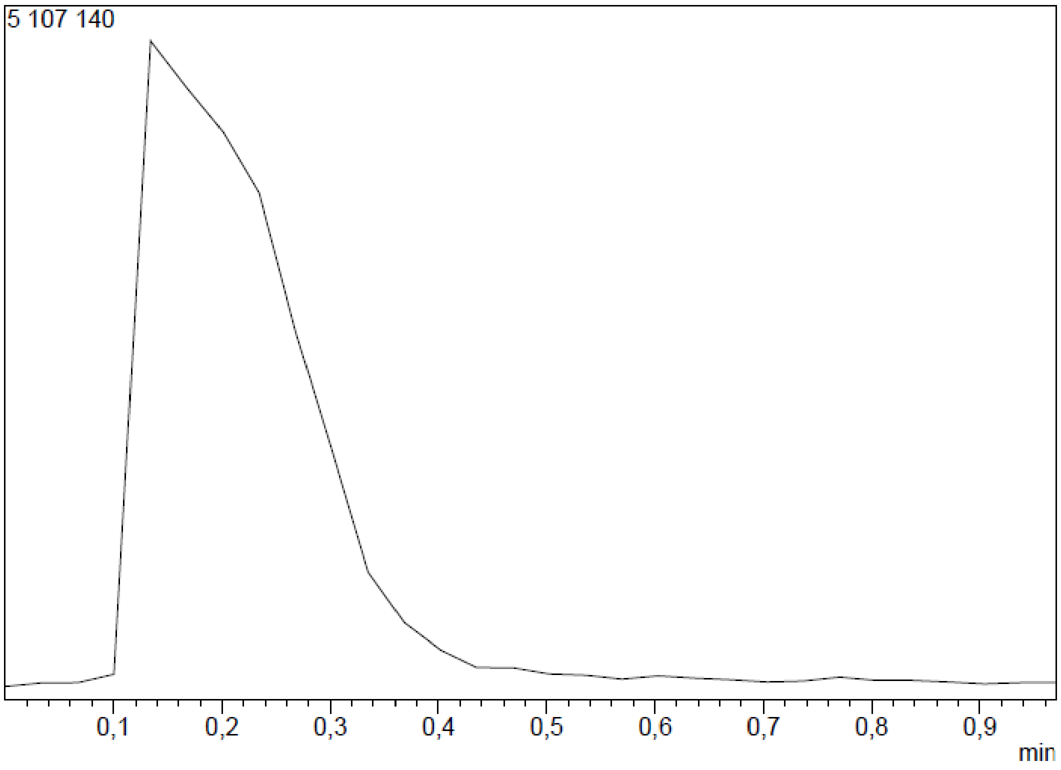

Figure S6 Chromatogram of Silibinin (m/z = 482.40) of ethanolic extract

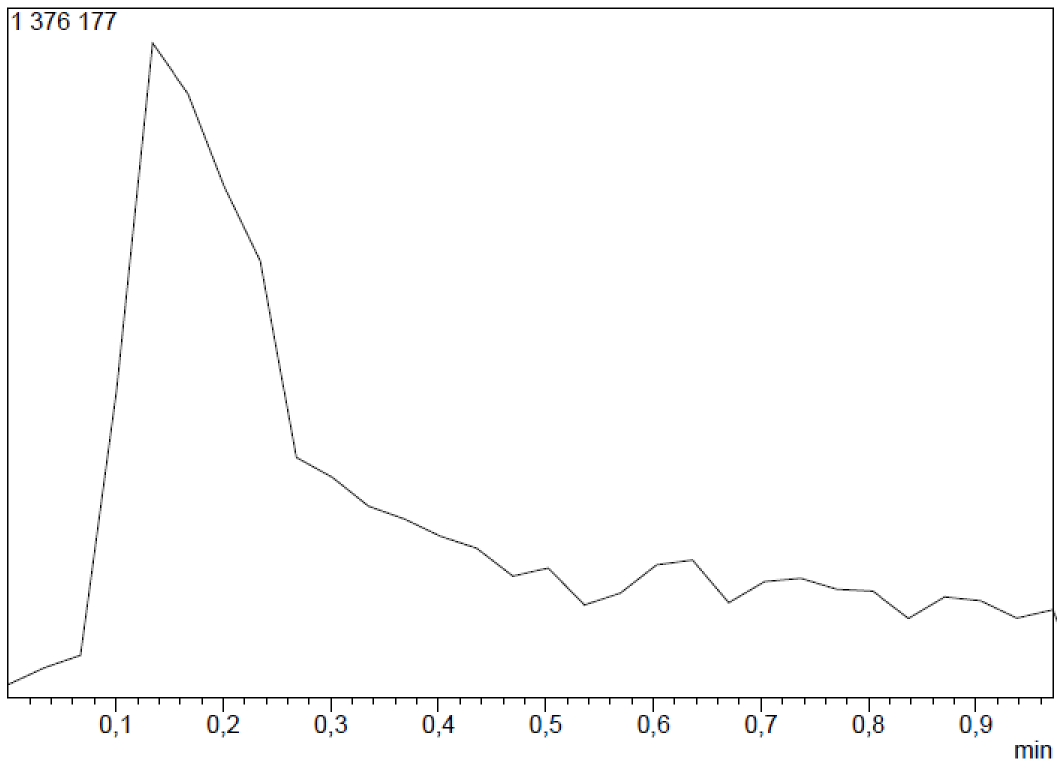

Figure S7 Chromatogram of Isosilybin A (m/z = 482.40) of ethanolic extract

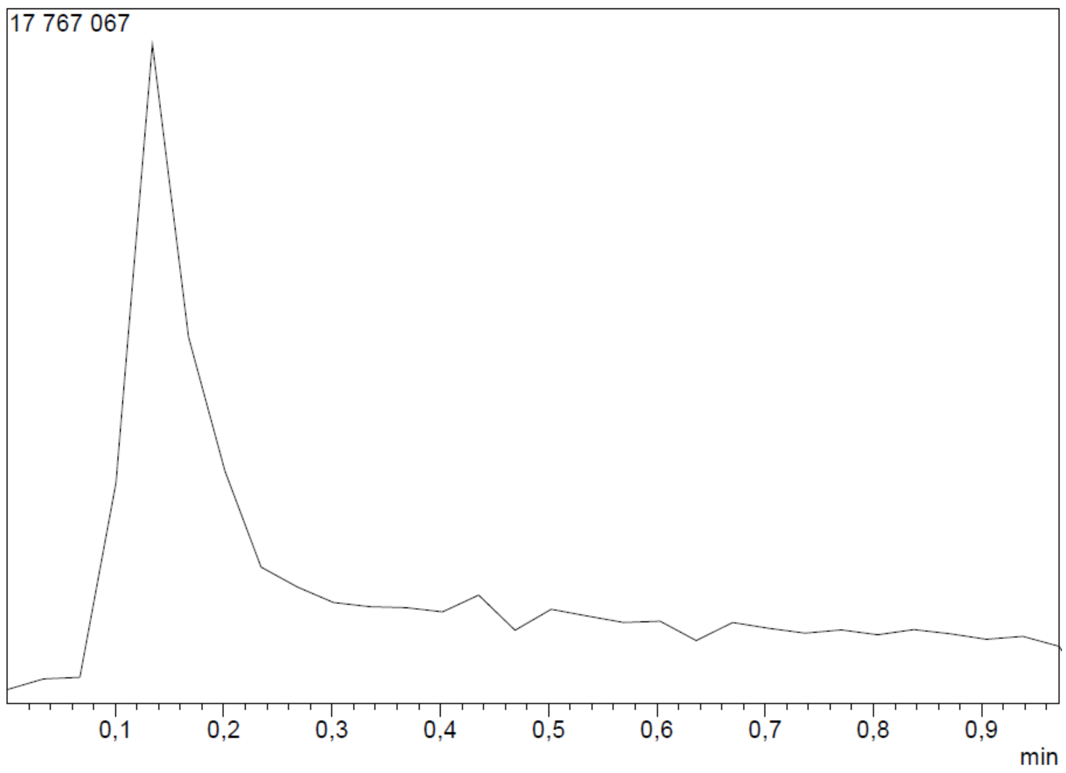

Figure S8 Chromatogram of Isosilybin B (m/z = 482.40) of ethanolic extract

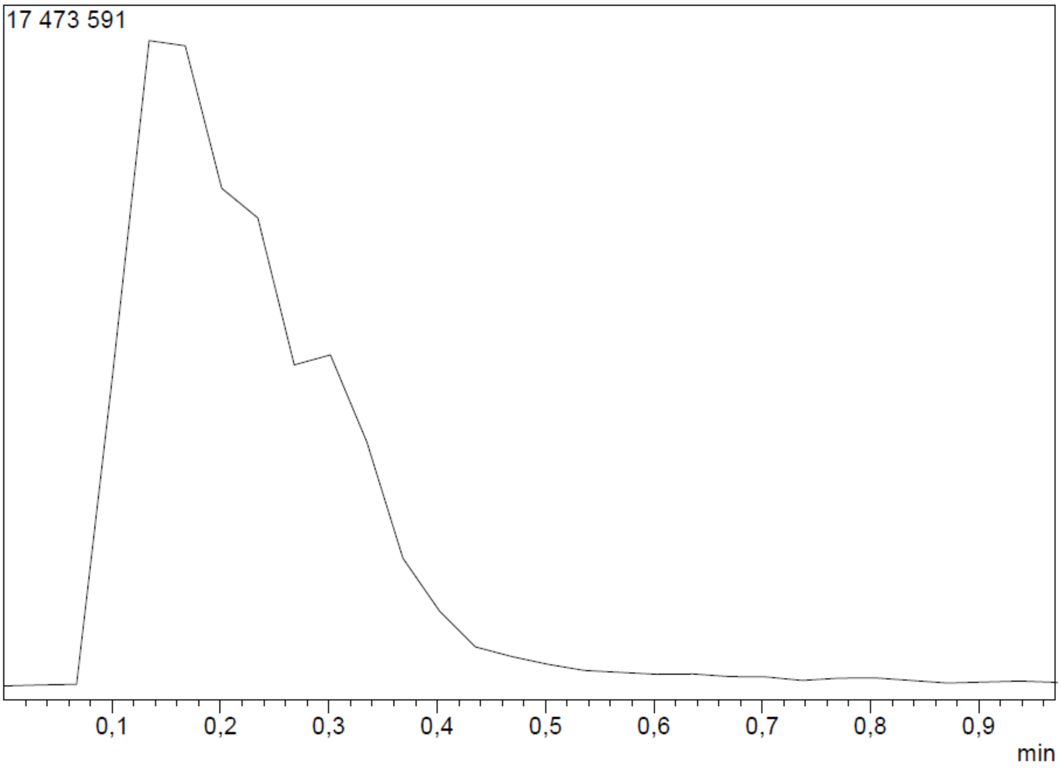

Figure S9 Chromatogram of Silychristin (m/z = 482.40) of ethanolic extract

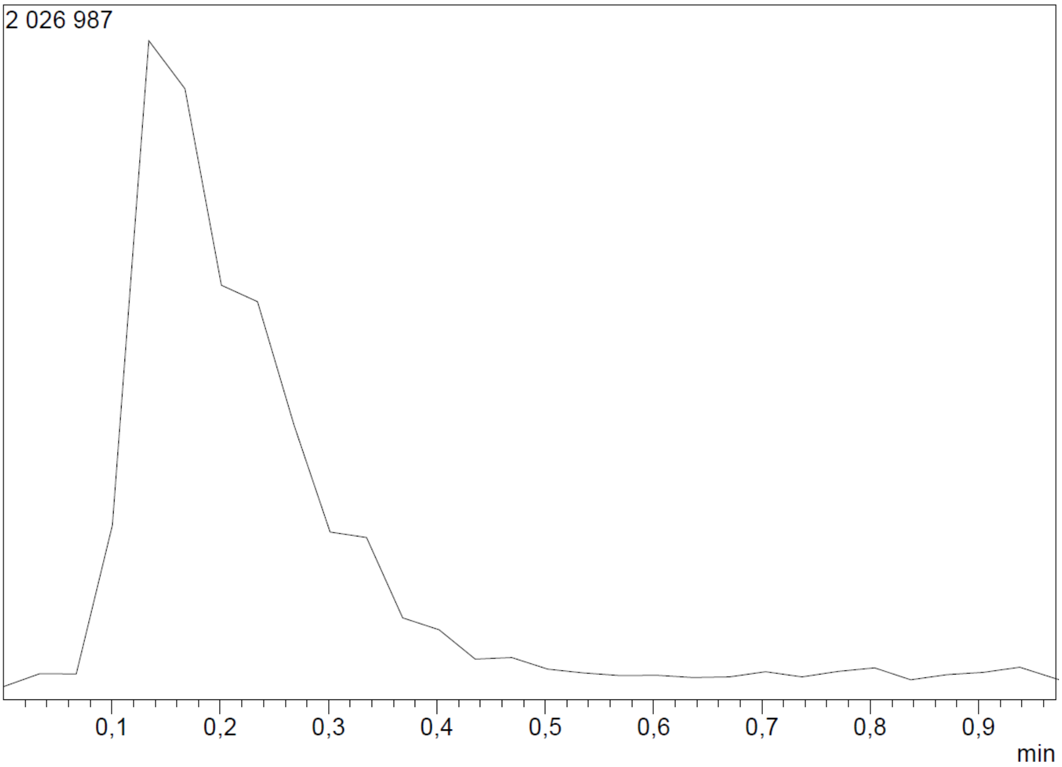

Figure S10 Chromatogram of Silydianin (m/z = 482.40) of ethanolic extract

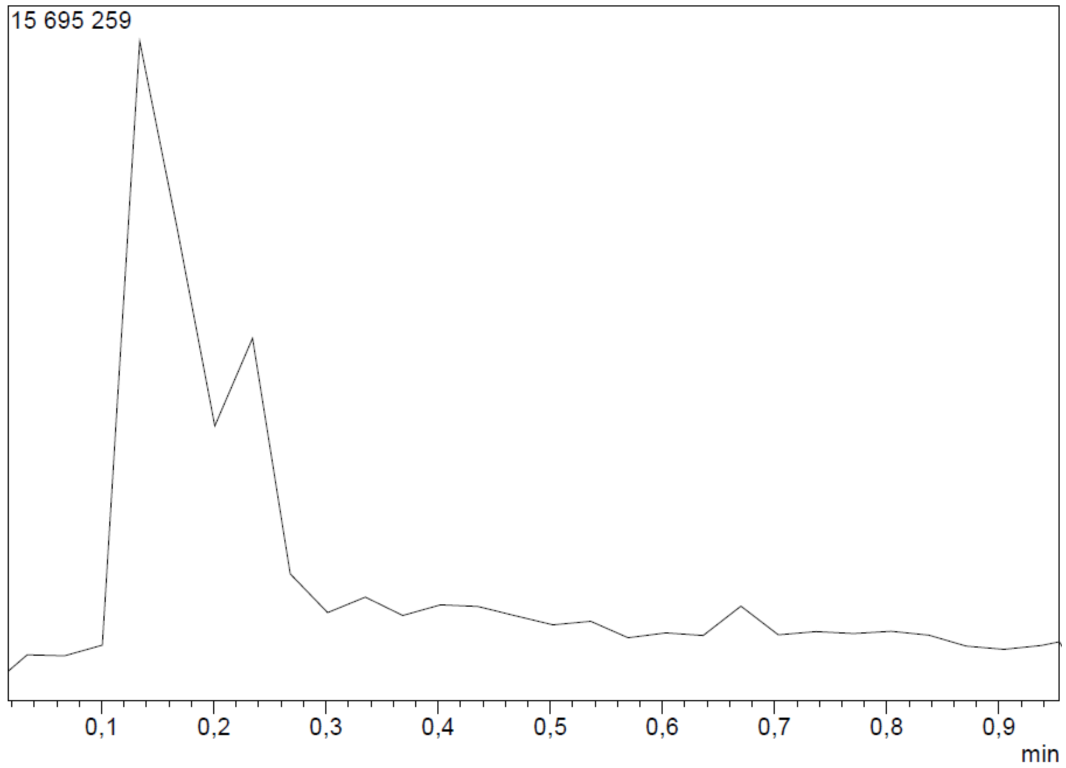

Figure S11 Chromatogram of Dehydrodiconiferyl alcohol (m/z = 358.40) of ethanolic extract

3. LC-MS/MS data: Methanolic extract of *Silybum marianum*

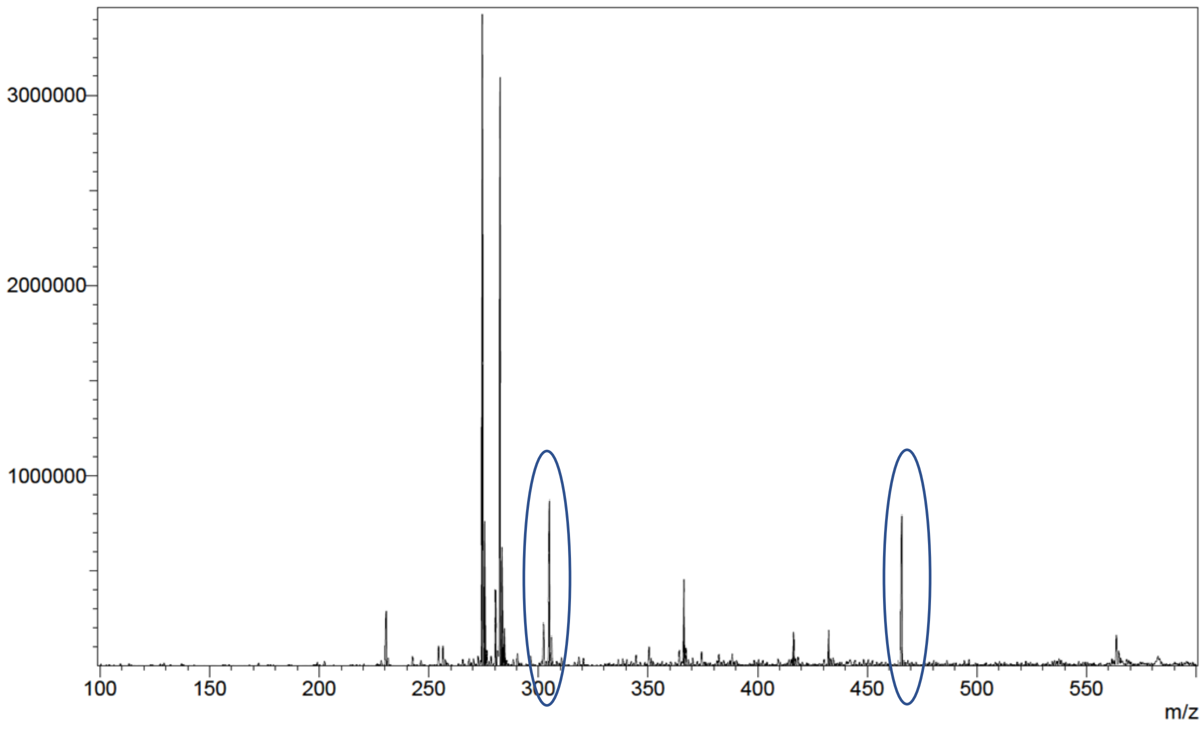

Figure S12 MS spectrum of methanolic extract

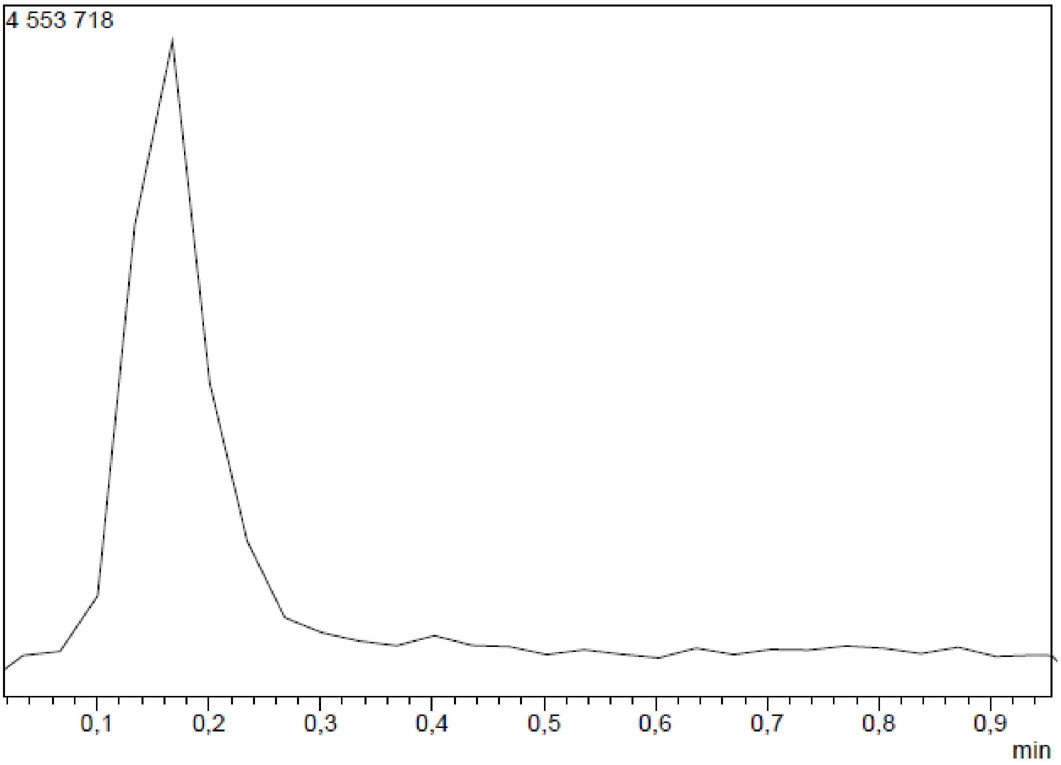

Figure S13 Chromatogram of Neosilyhermin A (m/z = 466.40) of methanolic extract

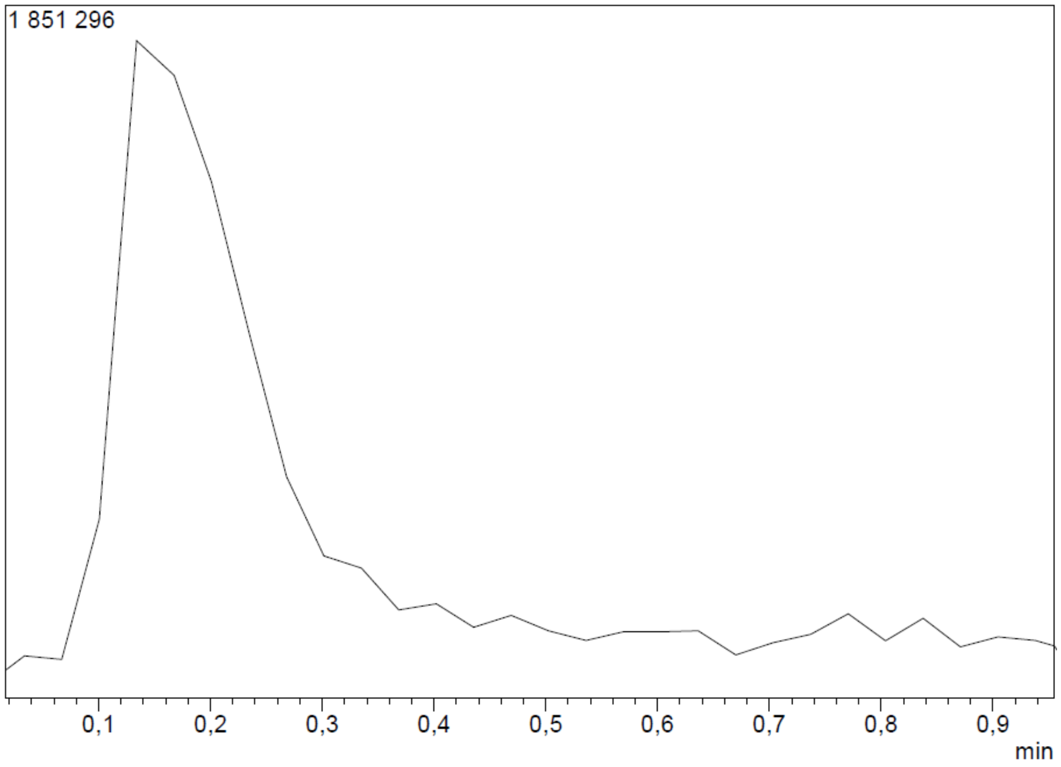

Figure S14 Chromatogram of Silyhermin (m/z = 466.40) of methanolic extract

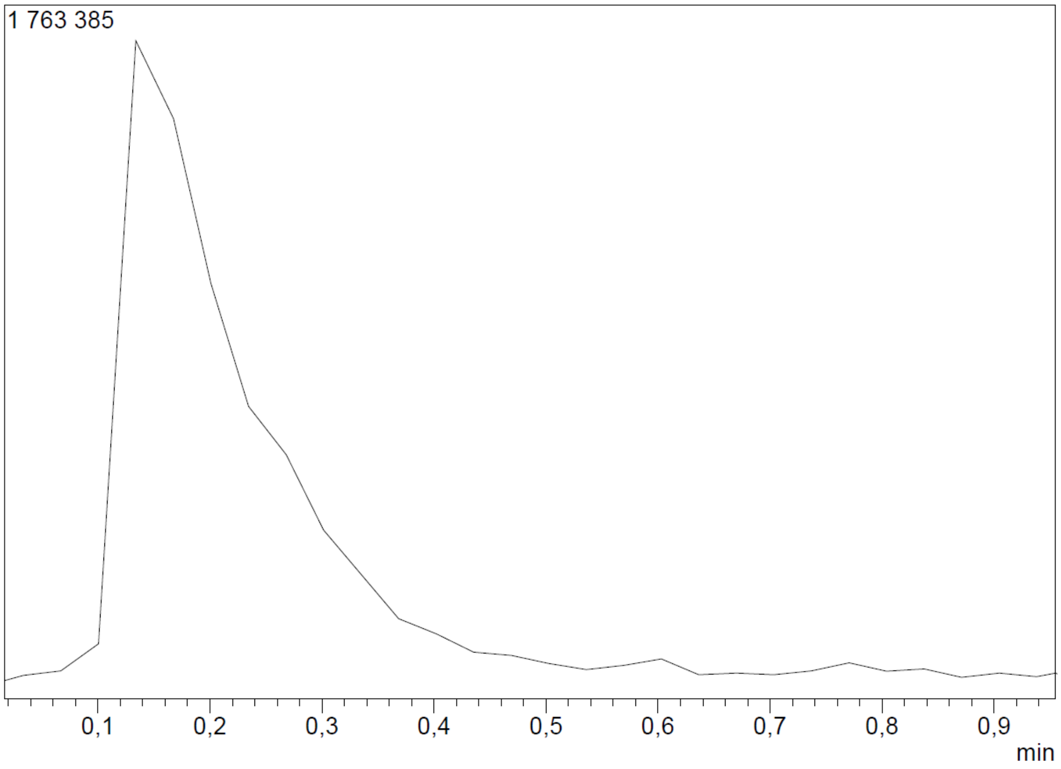

Figure S15 Chromatogram of Taxifolin (m/z = 304.25) of methanolic extract
